# The EU-TOPIA evaluation tool: An online modelling-based tool for informing breast, cervical, and colorectal cancer screening decisions in Europe

**DOI:** 10.1016/j.pmedr.2021.101392

**Published:** 2021-04-30

**Authors:** Andrea Gini, Nicolien T. van Ravesteyn, Erik E.L. Jansen, Eveline A.M. Heijnsdijk, Carlo Senore, Ahti Anttila, Dominika Novak Mlakar, Piret Veerus, Marcell Csanádi, Nadine Zielonke, Sirpa Heinävaara, György Széles, Nereo Segnan, Harry J. de Koning, Iris Lansdorp-Vogelaar, on behalf of the EU-TOPIA consortium

**Affiliations:** a Department of Public Health, Erasmus MC, University Medical Center Rotterdam, Rotterdam, the Netherlands; b SC Epidemiology, Screening, Cancer Registry, Città della Salute e della Scienza University Hospital, CPO, Turin, Italy; c Finnish Cancer Registry, Helsinki, Finland; d National Institute for Public Health, Ljubljana, Slovenia; e National Institute for Health Development, Tallinn, Estonia; f Syreon Research Institute, Budapest, Hungary

**Keywords:** Online screening evaluation tool, Miscrosimulation models, Cancer screening, Colorectal cancer

## Abstract

**Background:**

Aiming to support European countries in improving their breast, cervical, and colorectal cancer (CRC) screening programmes, the EU-TOPIA consortium has developed an online user-friendly tool (the EU-TOPIA evaluation tool; https://miscan.eu-topia.org) based on the Microsimulation Screening Analysis (MISCAN) model.

**Methods:**

We designed an online platform that allows stakeholders to use their country-specific data (demographic, epidemiological, and cancer screening information) to quantify future harms and benefits of different cancer screening scenarios in their country. Current cancer screening programmes and impacts of potential changes in screening protocols (such as extending target ages or increasing screening attendance) can be simulated. Results are scaled to the country-specific population. To illustrate the tool, we used the tool to simulate two different CRC screening scenarios in the Netherlands: biennial fecal immunochemical testing (FIT) in ages 55–75 and colonoscopy every ten years in ages 55–75. Data from the Dutch screening programme was used to inform both scenarios.

**Results:**

A total of 482,700 CRC cases and 178,000 CRC deaths were estimated in the Netherlands with FIT screening (for individuals aged 40–100 years, 2018–2050), with 47.3 million FITs performed (1.92 million positives of which 1.64 million adhered to diagnostic colonoscopy). With colonoscopy screening, CRC incidence and mortality were, respectively, up to 17% and 14% lower than in the current FIT screening programme, requiring, however, a colonoscopy demand that was 7-fold higher.

**Conclusions:**

Our study presents an essential online tool for stakeholders and medical societies to quantify estimates of benefits and harms of early cancer detection in Europe.

## 1 Introduction

Many of the current microsimulation models for cancer screening are proprietary, and open tools are only available for a handful of countries (Gauvreau et al., 2017), limiting the opportunity of policymakers to use such tools for decision-making. Moreover, using simulation models requires sufficient technical proficiency in understanding the model structure and the uncertainty behind the modelling analysis (Çağlayan et al., 2018). The Microsimulation Screening Analysis (MISCAN) model, a well-established microsimulation model used in informing cancer screening decisions worldwide (Knudsen et al., 2016, de Carvalho et al., 2017, Jansen et al., 2020a, Tomonaga et al., 2018, Sankatsing et al., 2020) is one of the leading tools for decision making in cancer screening. The EU-TOPIA project (EU-Framework Programme, Horizon 2020 – 634753) aims to share knowledge, create professional networks, and assist all European countries in monitoring, evaluating, and improving their screening programmes for breast cancer (BC); cervical cancer (CC); and colorectal cancer (CRC). Thus, we developed a user-friendly online tool based on the MISCAN model (EU-TOPIA Evaluation tool; https://miscan.eu-topia.org) to allow European stakeholders (i.e. policymakers, screening programme managers, coordinators, and researchers) to predict future outcomes of BC, CC and CRC screening scenarios in their countries. In this report, we describe this online tool and illustrate its use with an example for CRC screening.

## 2 Methods

### 2.1 The EU-TOPIA evaluation tool

The EU-TOPIA evaluation tool was developed for three cancer sites (BC, CC, and CRC) according to the same principles. In this report, we present a short introduction of the CRC version of the EU-TOPIA evaluation tool (with more detailed information of the CRC version in Supplementary Methods Part 1). The core structure of the CRC version of the tool was previously tested modelling the costs and benefits of the Hungarian FIT screening programme (Csanádi et al., 2020). Detailed information on the BC and CC versions can be found on the tool’s website. Application and results of the CC version of the tool were also reported in a previous publication (Jansen et al., 2021). The current version of the tool requires a registration (Supplementary Methods Part 1), and a direct approval is granted by the EU-TOPIA team to European stakeholders involved in the field of cancer screening.

### 2.2 The Colorectal cancer version of the EU-TOPIA evaluation tool

Four benchmark models form the basis of the CRC version of the EU-TOPIA evaluation tool, one for each region in Europe: a Dutch model version for Western Europe, a Slovenian version for Eastern Europe, a Finnish version for Northern Europe, and an Italian version for Southern Europe. The development and validation of those benchmark models is described in a previous publication (Gini et al., 2021). Briefly, the models were quantified based on local data on population demography (i.e. age distribution of the population and life expectancy), disease risk (i.e. CRC incidence, stage distribution and survival) and screening programmes (i.e. design of the screening programme, participation, positivity and detection rates). Subsequently, we externally validated three out of four models against the best available regional data concerning CRC screening effectiveness on CRC mortality reduction (no regional data on screening effectiveness was available for Eastern Europe), identified in a systematic review of the literature (Gini et al., 2020).

In our tool, stakeholders can download an Excel-template (Supplementary Methods Part 1: Supplementary Figs. 1–3) to provide country-specific data on their population demography, CRC epidemiology and CRC screening programme. The Excel-template includes a comprehensive overview of all data that needs to be collected to be able to effectively monitor and evaluate cancer screening programmes. Some data (so called “must have data”) are considered to be more important than other for obtaining long-term screening estimates that reflect a country’s situation, such as CRC incidence, the design of the screening programme and screening participation (see Supplementary Methods Part 1**:** Supplementary Fig. 4 and Supplementary Table 1 for a complete overview). For other data, if stakeholders are not able to collect accurate country-specific data (i.e. CRC relative survival, stage distribution, screening history, etc.), they can use data from the corresponding regional benchmark model. Specific information on the benchmark models can be retrieved in the cancer fact sheets available in the tool (Supplementary Methods Part 2). The more data the stakeholders provide for the tool, the better the tool will reflect the situation in their country.

Once the stakeholders have filled out the data template, they can upload it and specifically developed algorithms will check the quality and completeness of the uploaded data (see Supplementary Methods Part 1**:** Supplementary Fig. 6). Data that have passed the quality checks can be used to adjust the parameters in the benchmark model. Model parameters that will be automatically adjusted include: demographic parameters, such as size and age distribution of the population; natural history parameters, such as background risk of CRC and stage-specific relative survival; and screening parameters, including design of the programme, participation and test characteristics. Formulas used to adjust the parameters of the benchmark model can be found in the Supplementary Methods Part 3.

After the data check, the stakeholders can perform country-specific model simulations (Supplementary Methods Part 1**:** Supplementary Fig. 7). In addition to no screening and the current screening scenarios, they can choose alternative screening scenarios varying screening test, target age, screening interval, participation rates, or invitation coverage. Those options are dynamically incorporated in the model: changes are assumed to be implemented in 2018 (year of the first modelling EU-TOPIA workshop). Before 2018, screening is simulated according to the data uploaded by the user in the Excel data template. The tool predicts the number of cancer cases, cancer-specific deaths, screening tests, positive tests, diagnostic follow-up tests, and complications per calendar year (2018–2050). Model results are scaled to the country-specific population (Supplementary Methods Part 1: Supplementary Figs. 8–10). In addition, the tool includes a cost-effectiveness calculator that estimates lifetime costs and benefits of screening in the population (not illustrated in this report).

We started developing the tool in 2016. We presented it during the second, third, and fourth workshop of the EU-TOPIA project (Sweden, 2018; Italy, 2019; Belgium, 2020). A survey was conducted among the participants of those workshops (European stakeholders) and feedback was collected and used to update the tool.

### 2.3 Exemplary modelling analysis

Using the CRC version of the EU-TOPIA evaluation tool, we estimated the future CRC outcomes in The Netherlands, including expected CRC incidence cases, CRC deaths, and resources required by the CRC screening programme, such as number of screening tests, positive tests, and diagnostic colonoscopies. The Dutch national screening programme for CRC, with biennial FIT, was initiated in 2014. Among 741,914 persons invited for FIT, 529,056 (71.3%) participated (Toes-Zoutendijk et al., 2017). In our analysis, we compared three potential screening scenarios: i) no-screening; ii) the current screening strategy offered to the entire population in the age target (biennial FIT 47 µg Hb/g, from age 55 to 75, 71.3% participation rates); or iii) assuming colonoscopy screening as primary screening test (from age 55 to 75, every 10 years, 71.3% participation rates) after 2018 (before 2018, FIT was offered).

## 3 Results

### 3.1 The EU-TOPIA evaluation tool

During the EU-TOPIA workshops, the evaluation tool was used by 120 researchers, screening programme coordinators, and policymakers from 27 European countries. Among those, 78 completed the survey and 74% of them reported positive feedbacks.

### 3.2 Exemplary modelling analysis

**Fig. 1:**
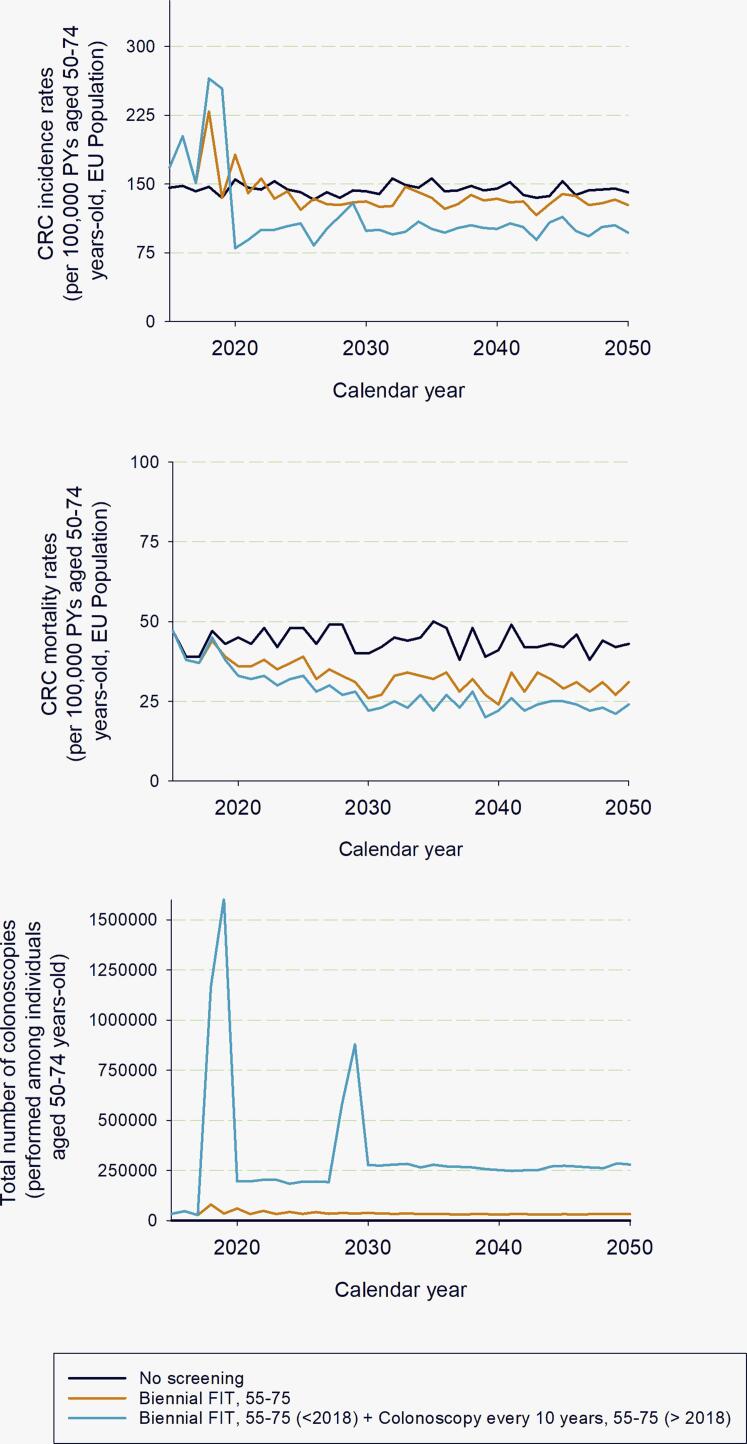
Colorectal cancer incidence, mortality rates, and total number of colonoscopies predicted per simulated scenario and calendar year. CRC = Colorectal cancer. Note: The total number of colonoscopies performed is composed by the total number of screening colonoscopy and the total number of diagnostic colonoscopies following a positive stool test result.

According to the EU-TOPIA evaluation tool using Dutch data, a total of 482,700 CRC cases and 178,000 CRC deaths were predicted in The Netherlands with biennial FIT screening from age 55 to 75 (for individuals aged 40–100 years; period 2018–2050; Table 1). Compared to no screening, CRC mortality was reduced by 27.5% among individuals aged 50–74 years-old (2018–2050; Fig. 1). This programme required 47.3 million FIT of which 1.92 million were positive and 1.64 million were followed by a diagnostic colonoscopy. Considering a change in the primary test in 2018 (colonoscopy every 10 years instead of biennial FIT), the predicted number of CRC cases and deaths decreased, respectively, to 401,100 (−17%) and 153,700 (−14%) among 9.1 million individuals aged 40–100 years, with 12.42 million colonoscopies (4 million positive tests) performed during 2018–2050, which was 7-fold higher than in the current screening programme (1.64 million) (Table 1). In addition, the number of complications (including serious, cardiovascular, and other complications such as nausea, vomiting, and abdominal pain) was 3-fold higher (58,880 with colonoscopy screening vs 18,500 with FIT). The detailed simulation report is presented in Supplementary Methods Part 4.

**Table 1:** Screening outcomes (×10,000) for each simulated scenario in individuals aged 40–100, 2018–2050.

	No screening	Current (Biennial FIT, age 55–75)	Colonoscopy (every 5 years, age 55–75)
Population older than 40 years in 2018	912.33	912.33	912.33
Person-years, 2018–2050	33118.9	33118.9	33118.9
Colorectal cancer cases (age 40–100)	56.49	48.27	40.11
Colorectal cancer incidence reduction (%,40–100) compared to no screening	–	14.56	29
Colorectal cancer deaths (age 40–100)	24.18	17.8	15.37
Colorectal cancer mortality reduction (%,40–100) compared to no screening	–	26.39	36.44
Colorectal cancer cases (age 50–74)	27.02	25.61	20.94
Colorectal cancer incidence reduction (%,50–74) compared to no screening	–	5.23	22.5
Colorectal cancer deaths (age 50–74)	8.87	6.39	5.58
Colorectal cancer mortality reduction (%,50–74) compared to no screening	–	27.92	37.1
Primary screening tests:			
- gFOBT	0	0	0
- FIT	0	4731.24	0
- FS	0	0	0
- Colonoscopy	0	0	1242.13
Positive screening tests	0	192.87	402.58
Diagnostic follow-up colonoscopies performed	0	164.01	–
False Positive (%)	0	2.27	0
Colonoscopy complications	0	1.85	5.88
Adenomas Detected	0	88.59	260.26
Colorectal Cancers Detected	0	9.8	5.65

gFOBT, guaiac fecal occult blood test; FIT, fecal immuchemical test (47 µg/gr); FS, Flexible-sigmoidoscopy.

## 4 Discussion

This study presents the EU-TOPIA evaluation tool, a user-friendly web-based cancer screening evaluation tool based on the MISCAN model. This online tool can be an effective and reliable resource for European policymakers that aim for informed decision-making on cancer screening in their country. By collecting data on CRC screening in The Netherlands, we were able to use the tool in a modelling example. We estimated that up to an additional 14% of CRC deaths might be avoided with colonoscopy screening (every 10 years, from age 55 to 75) in the Netherlands compared to the current biennial FIT screening scenario (same target age and participation rate). However, the endoscopy demand required by this colonoscopy scenario was 7-fold higher than with the current FIT scenario, resulting in a higher number of colonoscopy complications.

For policymakers, it is essential to get an insight into cancer screening’s favourable and unfavourable short- and long-term effects. Web-based cancer screening evaluation tools are scarce. Few have been developed to provide insights in cancer screening decision-making. Among those, the more relevant were provided within the CISNET modelling group, assessing the effectiveness of cancer screening in the US (van der Steen et al., 2015, Burnside et al., 2017). However, those tools do not allow stakeholders to upload country-specific data and obtain specific predictions for their local situation. The EU-TOPIA evaluation tool can be tailored to the local situation, but also permits the use of EU-TOPIA benchmark data when stakeholders do not have all the required information. We previously investigated which key priority data are essential to collect in monitoring cancer screening programmes (Csanádi et al., 2019), standardizing their definitions, criteria, and data templates throughout Europe. The EU-TOPIA evaluation tool is the natural consequence of that standardization work.

The burden of BC, CC, and CRC varies substantially among European countries (Bray et al., 2018). Thus, in developing our EU-TOPIA evaluation tool, we initially investigated those potential geographic differences, performing specific systematic reviews on the effectiveness of cancer screening in Europe. We found that the benefits of BC, CC, and CRC screening were quite consistent across European regions (Gini et al., 2020, Jansen et al., 2020b, Zielonke et al., 2020). Then, we analysed potential differences in the natural history of BC, CC, and CRC among a selected number of European countries, calibrating and validating 4 MISCAN model versions (The Netherlands, Italy, Slovenia, and Finland) for each cancer (BC, CC, and CRC). Those were used as benchmarks models: i) to take into account European regional differences inside the EU-TOPIA evaluation tool; and ii) to supply regional-specific data when stakeholders are unable to collect all data from their country. Developing and validating those regional model versions, we found that the differences in CRC incidence between countries could mainly be explained by differences in onset of disease, whereas the parameters behind the progression of disease could be assumed the same across the European regions (Gini et al., 2021). These last aspects were a strong support for the reliability and robustness of our CRC version of the tool.

Despite its strengths, the tool has some noteworthy limitations. First, simulations provided by the CRC version of the EU-TOPIA evaluation tool are not stratified by gender. Second, stakeholders can modify several model parameters uploading country-specific data, but some of those, such as participation in surveillance, surveillance protocol, or relative survival, could not be dynamically changed inside the tool. These could, however, be adjusted as part of the uploaded data template. Third, if screening performance indicators in a country vary meaningfully from the data and indicators used to structure the tool, this may limit and affect the reliability of the model estimates. Real-life monitoring and evaluation of cancer screening programmes therefore remains essential, especially considering the impacts of the COVID-19 pandemic on discontinuity of the programmes. There is remarkable uncertainty in the estimates on incidence and mortality impacts between some screening strategies (Buskermolen et al., 2019) and the modelled estimates depend respectively upon the natural history parameter values. Fourth, the EU-TOPIA evaluation tool is supported only up to the end of 2020 through funding (the EU-Framework Programme, Horizon 2020). It will remain available online, but future updates and support in interpreting the model results will be limited to European stakeholders involved in the field of cancer screening (their status will be assessed in the moment of the registration and access to the tool will be granted).

In conclusion, our study introduces an essential tool for helping policymakers and medical societies to inform their cancer screening decisions, combining both short-term (regular monitoring of quality-assurance performance) and long-term estimations. This tool may be crucial in quantifying future benefits and harms of early cancer detection in Europe and making future preventive programmes accountable to citizens and clinicians.

## Funding

Financial support for this study was provided entirely by the EU-Framework Programme (Horizon 2020, EU-TOPIA project, ref. number 634753, PI: HJ de Koning) of the European Commission. The funding agreement ensured the authors’ independence in designing the study, interpreting the data, writing, and publishing the report.

## Declaration of Competing Interest

The authors declare that they have no known competing financial interests or personal relationships that could have appeared to influence the work reported in this paper.
