# Supplementary material for: The EU-TOPIA evaluation tool: An online modelling-based tool for informing breast, cervical, and colorectal cancer screening decisions in Europe
Source: Prev Med Rep. 2021 Apr 30;22:101392. doi: 10.1016/j.pmedr.2021.101392 (PMC8122113; doi:10.1016/j.pmedr.2021.101392)
Supplement: Supplementary data 1 [file mmc1.docx]

**Supplementary Methods**

In this section, we report the online documentation related to the colorectal cancer version of the EU-TOPIA evaluation tool. This documentation is composed of four sections:

- EU-TOPIA evaluation tool user’s guide (part 1)
- Colorectal cancer fact sheets (part 2)
- Model adjustment based on the user’s data (part 3)
- Results of the Modelling example (part 4)

This documentation (except part 4) can be downloaded clicking the “information” icon in the dashboard (**Supplementary Figure 1**).
