# Supplementary material for: The EU-TOPIA evaluation tool: An online modelling-based tool for informing breast, cervical, and colorectal cancer screening decisions in Europe
Source: Prev Med Rep. 2021 Apr 30;22:101392. doi: 10.1016/j.pmedr.2021.101392 (PMC8122113; doi:10.1016/j.pmedr.2021.101392)
Supplement: Supplementary data 2 [file mmc2.docx]

**EU-TOPIA evaluation tool user’s guide**

**Account management**

Account creation

The web-tool is available at the following website (<https://miscan.eu-topia.org>). However, before using the model, you will need to register to the website and create a personal account. You can do this operation clicking “Register” (below the “Sign in” button) and filling the corresponding registration form. Before clicking the “Register” button on the bottom of the page, please be careful to have filled all the required information (fields labelled with *). Clicking the “Register” button, your registration form will be sent to the system and you should receive a first confirmation e-mail, testing the correctness of e-mail contact that you have provided. Please read carefully the test e-mail and click the link to confirm the correctness of your e-mail details.

After confirming that information, you will receive a final confirmation e-mail reporting that your application has been successfully sent to the website’s admins for the final authorization.

When your personal account has been approved by the admin, you will receive an approval e-mail and you can log in in the web-tool. The approval will be direct for stakeholders, policymakers, or researcher involved in the field of cancer screening in Europe.

Log in

When your account has been approved, you will be able to proceed with the log in. You may log in from the evaluation tool website (<https://miscan.eu-topia.org>) using your e-mail and the password that you defined during the registration process. When you log in the first time, you will be asked to download this user’s guide and to declare to have read it. After logging in, you will have access to the dashboard of the evaluation tool (**Supplementary Figure 1**).

Log out

You can log out from the application in anytime clicking the button “Logout” at the top right corner of the web site.

Change Password

You can view your account information and/or change your password clicking the link behind your name (top-right corner of the website, on the left of the logout button) or clicking on “My account”.


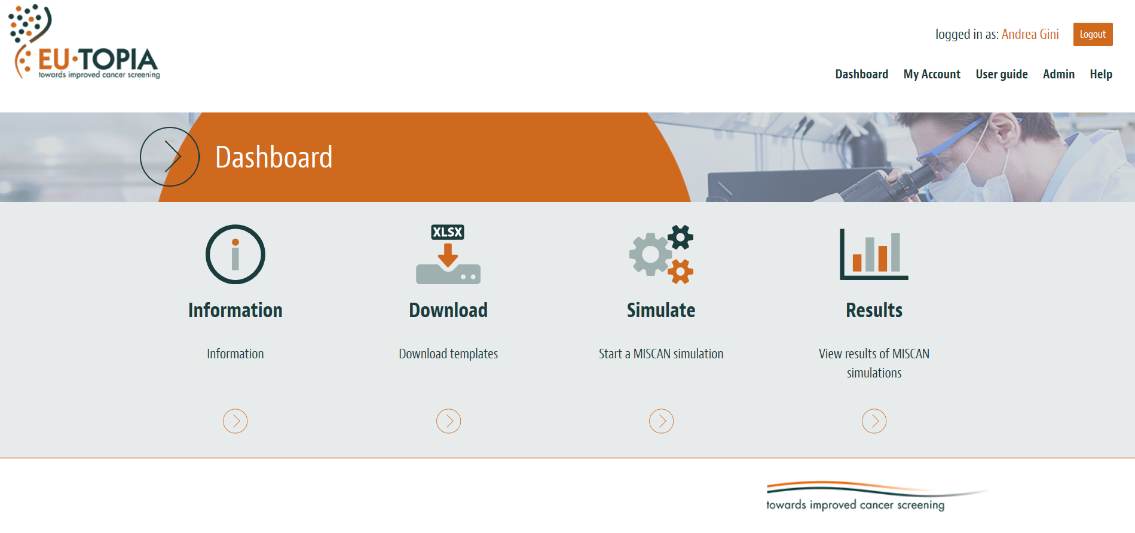


**Supplementary Figure 1**. EU-TOPIA evaluation tool, user’s dashboard.

Help

If you need any help, please feel free to email the EUTOPIA research team at [eu.topia@erasmusmc.nl](mailto:eu.topia@erasmusmc.nl) or click help (below the logout button) and fill in the help form (this last way will be very appreciated).

**Data Collection**

The MISCAN web-based tool was designed to allow users to simulate outcomes and harms of several cancer screening strategies for their own country. Therefore, this tool requires users to upload specific demographic and screening data for their own country using the provided data templates. This section of document describes how to download and fill in the colorectal cancer data templates.

Download Excel Data Templates

1. Log on to the main dashboard page.

Once you log in you will see the main evaluation tool dashboard (**Supplementary** **Figure 1**).

1. Click on “Download”.
2. You can download templates for each cancer site and save these on your computer (see **Supplementary Figure 2**). For the specific cancer template, click the cancer icon.


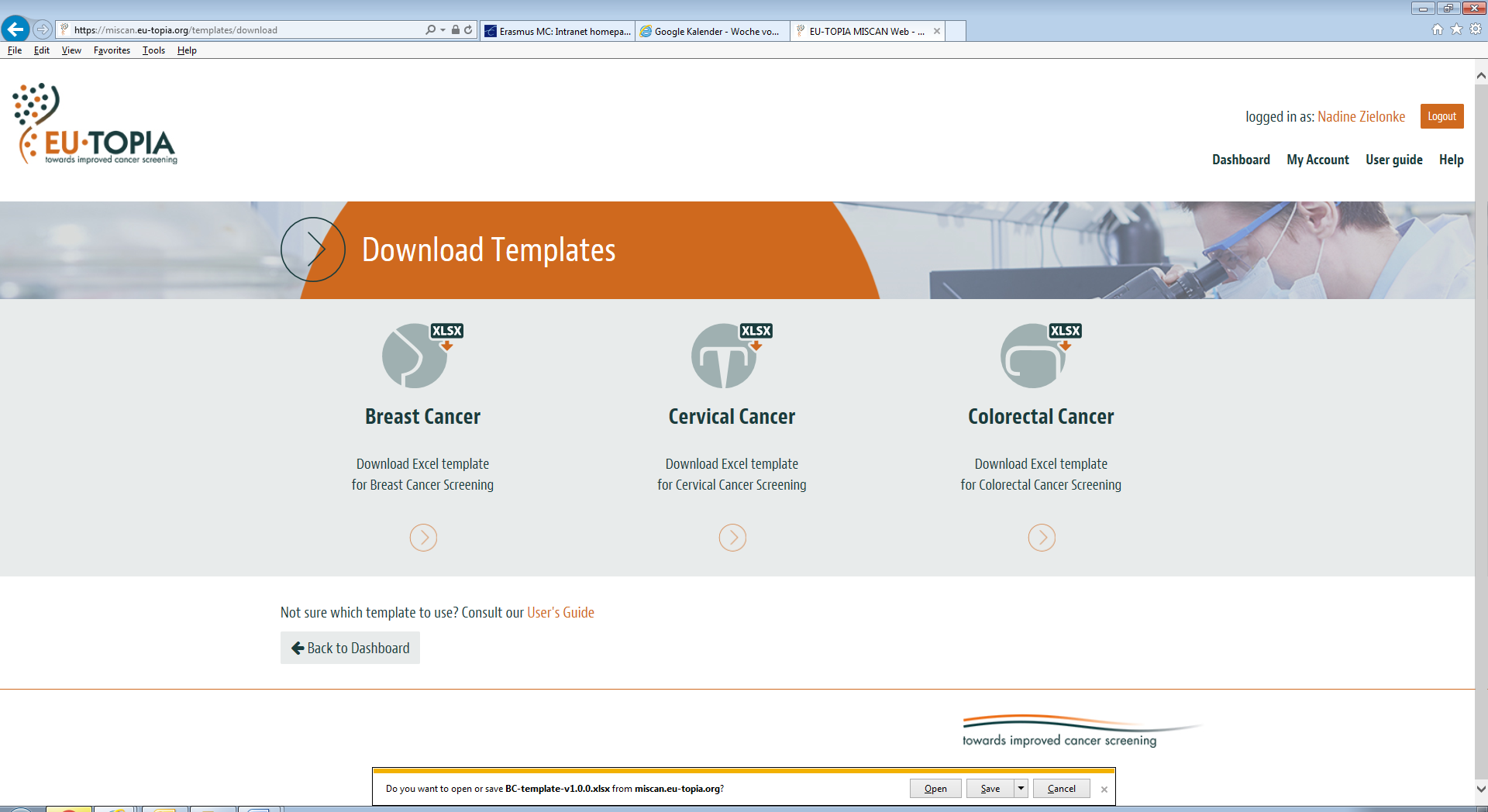


**Supplementary Figure 2**. EU-TOPIA evaluation tool, download templates section.

1. Save the templates to your computer by clicking “Save as” in Save option list (see **Supplementary** **Figure 3**)


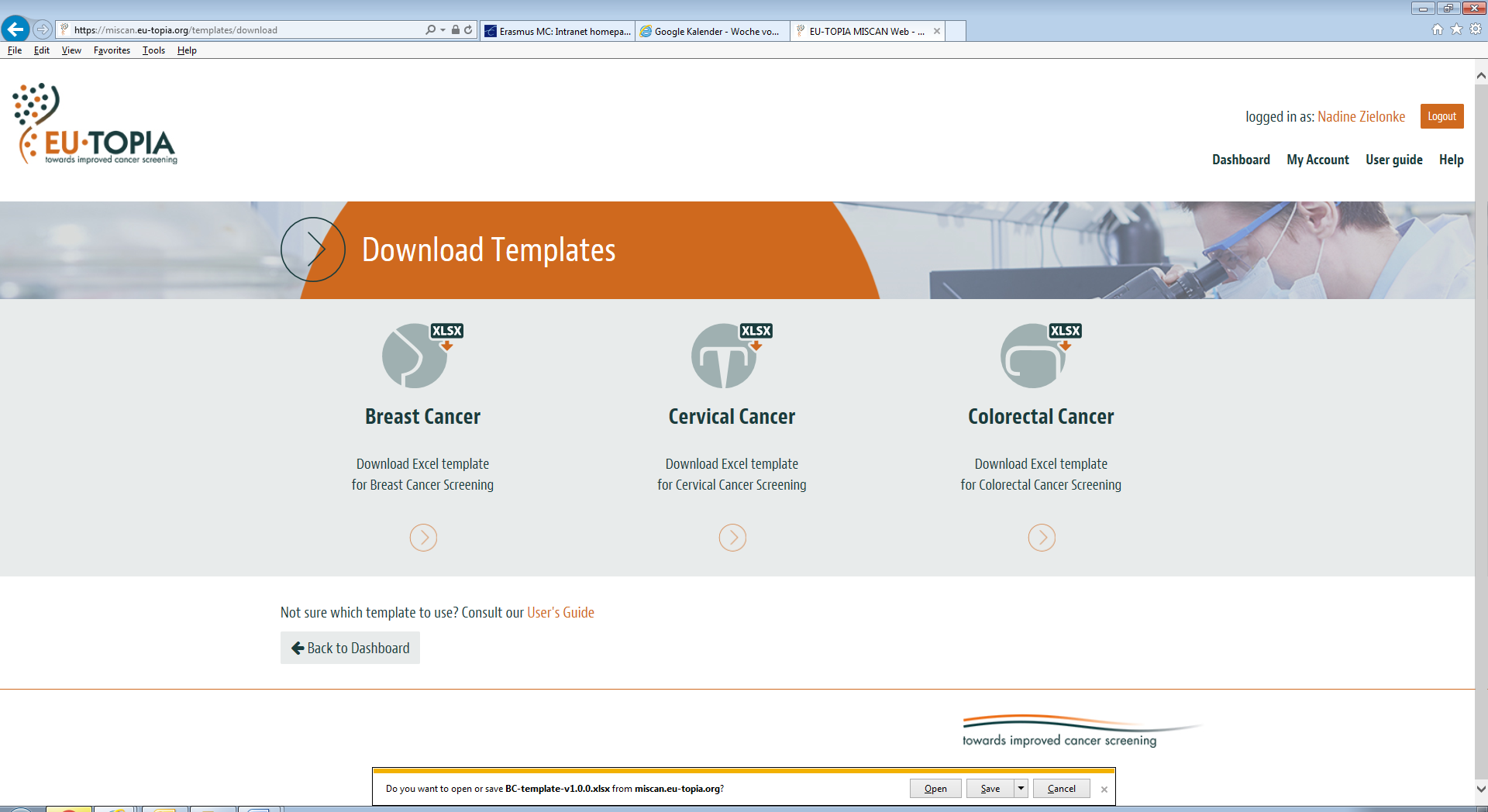


**Supplementary Figure 3**. EU-TOPIA evaluation tool, saving excel data template.

**Colorectal Cancer**

For simulating colorectal cancer screening benefits, harms, and costs in your country, the model requires country specific information.

To tailor the model to your own country, you need to upload the following information:

- population and epidemiological data (7 tables)
- screening monitoring information (15 tables)

A core set of tables is required for the model to run your simulations. These are labelled as ‘must have’ in **Supplementary Table 1**. Including information labelled as ‘should have’ or ‘nice to have’ will improve the quality of the information the model generates for your country. The tables used in this tool are an updated version of the tables designed for the report on the implementation of the council recommendation on cancer screening (Cancer screening in the European Union, 2017).^13^ Detailed information on how to fill those tables are provided in the appendix of that report and in the EU-TOPIA evaluation tool’s guide line (<https://miscan.eu-topia.org>). In this appendix, we reported detailed information on the tables not present in that report (please see in **Supplementary Table 1**). Data labelled as “required for the screening monitoring”, it is required for an external EU-TOPIA monitoring tool and will not be used inside the EU-TOPIA evaluation tool.

**Essential information for filling out the data tables:**

**Key instructions**

- Fill out the tables in the order that they are presented in the excel template.
- ‘Must have’ data is necessary to have a good refinement of the model for the country-specific analysis
- The more accurate and complete the country-specific data that you are providing, the more accurate and country-specific will be the results of the simulations.
- Fill only the white cells in the data templates. All other cells are non-editable.
- Only use the data type specified by the information box when you click on an empty cell indicated.
- Be careful with copying and pasting data from other Excel files. If data is pasted into non-editable cells, the tool will not recognize the inputs.

**Information about data quality**

- After you submit the data, automatic data quality checks will be applied to make sure the inputs fall within a reasonable range.
- Missing information can be dealt with by using data already provided by the EU-TOPIA research group (available for some countries). The more complete the input data, the better the model will simulate your scenarios.

**Trouble shooting**

- Make sure you always work with the most recent version of the user guide (download from <https://miscan.eu-topia.org>) as we are constantly updating this document to answer as many questions as possible.
- If you encounter any problems when entering the data, please contact the EU-TOPIA research team at: [eu.topia@erasmusmc.nl](mailto:eu.topia@erasmusmc.nl).

**Supplementary Table 1**. Overview of all data requirements for the EU-TOPIA web-tool for colorectal cancer.

| **Table name** | **Brief description** | **Level of**  **importance** | **Information already defined in the report (Cancer screening in the European Union, 2017)** |
| --- | --- | --- | --- |
| Table0 | Select your country | Must have | No |
| eTable1 | Population data | Must have | No |
| eTable2 | Cancer incidence | Must have | No |
| eTable3 | Cancer mortality | *Nice to have** | No |
| eTable4 | Relative survival by stage | Must have | No |
| eTable5 | Stage distribution | Must have* | No |
| eTable6 | All-cause mortality by age | Must have | No |
| eTable7 | Cancer localization distribution | Must have | No |
| sTable1a | Programme characteristics | Must have | No |
| sTable1b | Target population | Must have | Yes |
| sTable2 | Screening coverage | Must have | Yes |
| sTable3a | Screening history | Should have | Yes |
| sTable3b | Opportunistic screening coverage | *Nice to have** | Yes |
| sTable4 | Further assessment | Must have | Yes |
| sTable5 | Follow-up colonoscopy participation | Should have | Yes |
| sTable6a | Follow-up colonoscopy completion | Should have | Yes |
| sTable6b | Screening colonoscopy completion | Should have | Yes |
| sTable7a | Screening outcome  (gFOBT, FIT, or Colonoscopy) | Must have | Yes |
| sTable7b | Screening outcome  (Flexible Sigmoidoscopy) | Must have | Yes |
| sTable8 | Staging of screen detected cancers | Required for screening monitoring | Yes |
| sTable9 | Complications | Nice to have | Yes |
| sTable10 | Management recommendations | Required for screening monitoring | Yes |
| sTable11 | Interval cancers | *Should have** | Yes |

* In case of the Colorectal cancer version of the EU-TOPIA evaluation tool, those data are not used. However, those are required for the breast and cervical cancer version of the tool.

**Country**

Table0: Country

If you submit national data and not regional data, select your country from the drop-down menu (Country menu, **Supplementary Figure 4**). In this case you can ignore the second drop-down menu (Region). If your country is not listed, please contact us at: [eu.topia@erasmusmc.nl](mailto:eu.topia@erasmusmc.nl)

If you want to submit regional instead of national data, please contact us as at [eu.topia@erasmusmc.nl](mailto:eu.topia@erasmusmc.nl). After we assigned you to an index region, you need to select your country from the first drop-down menu (Country menu, Supplementary Figure 4) and the respective index region (A-Z) from the second drop-down menu (Region menu, **Supplementary Figure 4**).


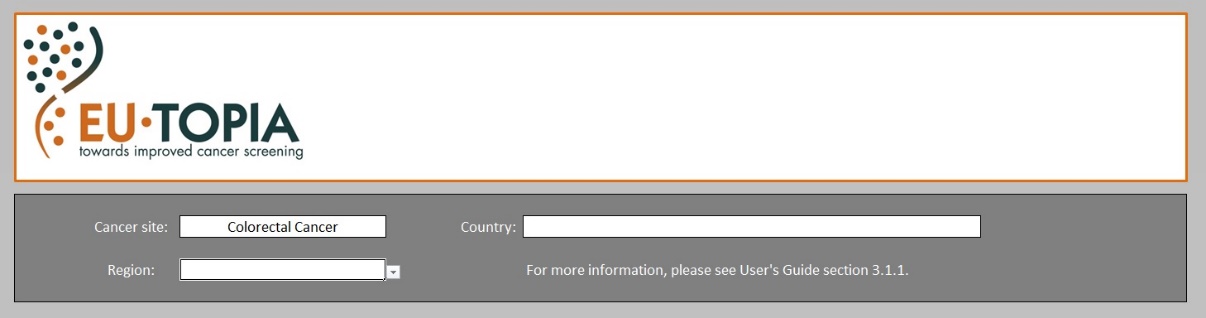


**Supplementary Figure 4**. EU-TOPIA MISCAN web-tool, Select your country (and region) in Table0.

Please be aware that we strongly advise you to use the same reference population in all tables!

**Epidemiological Data**

All tables for demographic and epidemiological data are marked as “eTablex”.

Please fill out all of them for your country. In this section, we show you how to fill them out correctly.

eTable1: Population age composition

| Level of importance | Must have |
| --- | --- |
| What does this table contain? | The population age distribution in the current year and population projections (up to 30 years ahead) for your country |
| Format | Separated by sex, calendar year and five-year age groups |
| Potential data sources | We suggest you use the base case scenario (i.e. the scenario based on current population trends). If there are several sources for population data in your country, we suggest using the source which is also used for official government projections. A good resource is EUROSTAT population projections  (<http://ec.europa.eu/eurostat/data/database>). |

eTable2: Colorectal cancer incidence rates

| Level of importance | Must have |
| --- | --- |
| What does this table contain? | The number of incident colorectal cancer cases (ICD-10: C18-C20) and person years at risk over the most recent five-year period available prior to introduction of screening. |
| What does this table NOT contain? | Carcinoma in situ cases are excluded from this table |
| Format | Separated by sex and five-year age groups |
| Potential data sources | National cancer registries |
| Trouble shooting | “Five-year period available prior to introduction of screening” indicates the most recent available period before introduction of screening in your country (i.e. Screening started in 2008, the most recent available period prior to screening should be 2003-2007).  If your estimates are labeled as “low quality data” in the web-based tool we recommend using a longer time period in reporting incidence data (10 most recent years instead of 5).  If you do not have direct access to national cancer registry data or detailed data are not available, we suggest checking the availability of cancer incidence data from the IARC: “Cancer in five continents dataset” (CI5, <http://ci5.iarc.fr/CI5I-X/Pages/download.aspx>)  If no cancer registry data is available for your country, we suggest using available cancer registry data from another, similar country. |

eTable3: Colorectal cancer mortality rates

| Level of importance | Should have |
| --- | --- |
| What does this table contain? | Mortality due to colorectal cancer (ICD-10: C18-C20) most recent five-year period available prior to introduction of screening. |
| What does this table NOT contain? | Carcinoma in situ cases are excluded from this table |
| Format | Separated by sex and five-year age groups |
| Potential data sources | National cause of death register |

eTable4: Colorectal cancer relative survival

| Level of importance | Must have |
| --- | --- |
| What does this table contain? | Probability of surviving at least 5 years after a diagnosis of colorectal cancer (ICD-10: C18-C20), observed in the five most recent years. |
| What does this table NOT contain? | Carcinoma in situ cases are excluded from this table |
| Format | Separated by sex and stage (TNM Classification of Malignant Tumors – UICC, <https://www.uicc.org/resources/tnm>). |
| Potential data sources | National cancer registries |
| Trouble shooting | Only numbers between 0 and 100 can be used as input (i.e. no words, notes, or symbols): typing 0.2, the web-tool will read 0.2%; and typing 20, 20%. Survival probabilities that show greater survival in advanced stages compared to lower stages will be marked as “low-quality” data in the web-based tool (please see section regarding “Quality Checks”) and you will be advised not to use this data when using the web-tool.  If your estimates are labeled as “low quality” or detailed data are not available, we recommend to check published data from EUROCARE group (<http://www.eurocare.it/>) |

eTable5: Colorectal cancer stage distribution

| Level of importance | Must have |
| --- | --- |
| What does this table contain? | Stage distribution of colorectal cancer (ICD-10: C18-C20) in the five most recent years available prior to introduction of screening. |
| What does this table NOT contain? | Carcinoma in situ cases are excluded from this table |
| Format | Separated by sex and stage (TNM Classification of Malignant Tumors – UICC,  <https://www.uicc.org/resources/tnm>). |
| Potential data sources | National cancer registries |
| Trouble shooting | If your stage distribution adds up to more than 100% (Percentage), the web-tool will mark your data as “low-quality” data. In that case please contact the EU-TOPIA research group at:  [eu.topia@erasmusmc.nl](https://miscan.paronix.nl/help). |

eTable6: Population all-cause mortality

| Level of importance | Must have |
| --- | --- |
| What does this table contain? | Current all-cause mortality rate |
| Format | Separated by sex and single ages (0-100) |
| Potential data sources | Life tables from national statistical offices |
| Trouble shooting | You are only allowed to input numbers (no words, notes, or symbols) between 0 and 1. Please make sure that values are not multiplied by 100,000 person-years (hence, values need to be reported considering 1 person-year).  The input needs to be age-specific mortality rates, not age-specific probabilities of death. In the projections of the web-tool, the all-cause mortality will be assumed to be stable over time. As alternative data we recommend your country’s life tables from the Human Mortality database  (<http://www.mortality.org/>, use the ‘mx’ column in the data provided). |

eTable7: Colorectal cancer localization

| Level of importance | Must have |
| --- | --- |
| What does this table contain? | Localization distribution of colorectal cancer (ICD-10: C18-C20) in the five most recent years prior to screening available prior to introduction of screening. |
| Format | Percentage values, separated by sex |
| Potential data sources | Studies in the average risk population |
| Trouble shooting | Only numbers between 0 and 100 can be used as input (i.e. no words, notes, or symbols): typing 0.2, MISCAN web-based tool will read 0.2%; and typing 20, 20%. |

**Screening Data**

All tables for screening data are marked as “sTablex”. Please fill out all of them for your country.

**General Instructions**

All tables would require data stratified by gender. If you cannot provide data by gender, please only fill the tables for men with your data with the data for entire target population

**Initial/subsequent tests**

Data in tables 3a, 4, 5, 6a, and 7a (tables of the excel template) on number individuals should be stratified by initial versus subsequent tests, if the programme(s) are population-based:

**Initial screening:**

For individuals for whom it is the first screening examination within the screening programme, regardless of the organizational screening round in which the examination takes place. Include also individuals who had screening tests or examinations performed in a population-based screening programme before the first invitation is received (these examinations are often referred to as “spontaneous tests”).

- **Subsequent screening:** For individuals with a screening examination within the screening programme following an initial screening examination, regardless of the organizational screening round in which the examination is performed.
- **Unknown if initial or subsequent screening:** The “Screening history is unknown” column (if present) should be used to enter the data of screened individuals for which the above distinction is not available.

The numbers collected in the three sub-tables should refer to strictly distinct sets of people. Always check the total figures at the bottom of the three tables to be sure that the sum of the strata is the total number expected.

sTable1a: Current Screening information

| Level of importance | Must have |
| --- | --- |
| What does this table contain? | Information on current screening strategy |
| Format | This data template collects several information:   - Historic screening information; - Current national screening strategy,   Stratified by age, screening test, and interval  (only two not parallel age groups are allowed);   - Referral and positivity test criteria; - Post-colonoscopy recommendations.   For formats, please check the box’s information included in each editable cell. |
| Potential data sources | National screening reports |
| Data definition and  Trouble shooting | **Definitions:**  Starting date of the programme  (Year when screening was introduced in your country)  End of the roll-out phase  (if roll-out completed before the index year).  Years needed for reaching the completed roll-out:  (No. of years needed from starting screening and completing roll-out).  Colonoscopy referral criteria – sigmoidoscopy (FS)  If sigmoidoscopy has been adopted as primary screening test in your programme, please indicate the criteria adopted for colonoscopy referral (i.e. positive sigmoidoscopy). You can indicate all the options which are applied in your programme (multiple answers allowed).  Positivity cut-off gFOBT/FIT  Please indicate the criteria for referring subjects for colonoscopy (i.e. positivity cut-off).  Post-colonoscopy surveillance  Please indicate the type of post-polypectomy surveillance protocol (test, interval and duration) for subjects by index lesion (most advanced lesion per person).  **Trouble shouting:**  Screening heterogeneity:  If in your country screening is implemented differently at regional level, please fill the data template with regional information or provide in this table the screening strategy that most represents your country.  No information of screening history:  If colorectal cancer screening was not introduced in the country before 2018, please insert 2018 in cell of “year of screening introduction”. It may be possible to declare a desire stratified screening strategy in the “Current national screening strategy” table. This may be simulated in the web-tool (from 2018 in forward as benchmark). If no “current national screening strategy” is provided, web-tool inputs Dutch national screening as default benchmark strategy (Biennial FIT from age 55 to age 75).  Current national screening strategy not age-stratified:  Please define country specific national screening strategy only using options provided for group 2.  Post-colonoscopy recommendations, referral, or positivity test criteria:  If information is not available, these cells may be left empty. In that case, web-tool inputs as default information already provided by the EU-TOPIA research group for specific exemplary country (in the web-tool will be possible to select which information user might use).  Important note:  If screening is not implemented uniformly across the country or region on which you are reporting (i.e. there is regional variation in the rollout of screening or there is regional variation in the eligible age range or frequency) please report a screening policy which best represents the most common policy in your country/region.  If you are in doubt, please contact us at: [eu.topia@erasmusmc.nl](mailto:eu.topia@erasmusmc.nl). |

**Simulation**

In this section of the document you will find instructions on how to use the web based MISCAN tool to simulate different colorectal cancer screening strategies for your country.

After registering and filling out the excel data templates, as described in above, you are ready to start a simulation.

Upload data

Log in into the EU-TOPIA evaluation tool (<https://miscan.eu-topia.org/login>) with your e-mail and password. Go to the simulation section the MISCAN web- tool dashboard. Start by selecting the cancer type, colorectal cancer in this case, and giving your simulation a name. (**Supplementary Figure 5**).


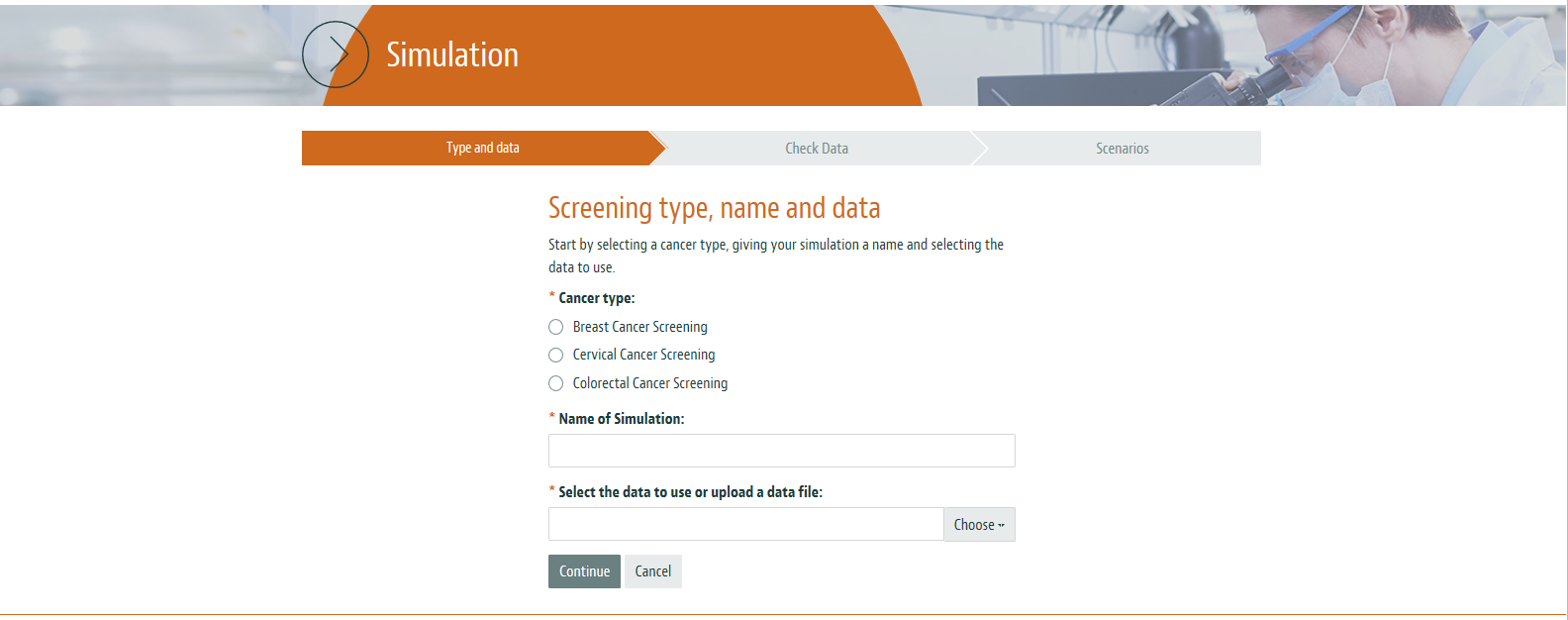


**Supplementary Figure 5**. EU-TOPIA evaluation tool, Screening type, name and data

Then, upload the data for your country by clicking “Choose file” and select the data template that you completed as described above. Users from one of the exemplary countries (Finland, Italy, the Netherlands and Slovenia) can click on the “Choose” button and either directly select the data from their country in the drop-down menu or select “upload your own data” to upload the data template. Press “Continue” (**Supplementary Figure 5**).

Quality check of the data

After uploading your data, the web-based tool performs checks on data quality and completeness. In the next step you are asked to verify your data (**Supplementary Figure 6**). If you wish you can choose to use data already provided by the exemplary country of your region for non-mandatory data (North: Finland, South: Italy, West: the Netherlands, East: Slovenia). If you think that data of the exemplary country of your region might not provide a reliable solution for your simulations and you prefer to use data already provided by the exemplary country of another region, please contact us at: [eu.topia@erasmusmc.nl](mailto:eu.topia@erasmusmc.nl). If the data that you uploaded is missing, incomplete or of insufficient quality for specific non-mandatory tables, the tool will automatically select the option to use the respective data from the exemplary country.

If you did enter data in the template but the evaluation tool marks your data as insufficient quality, please check if the format of your data is as specified above (or in the appendix of the 2017 European Report on cancer screening),^13^ within the allowed limits and proportions add up to 100% if applicable. Also, read the ‘trouble shooting’ part at the bottom of the respective instruction table if present. Cost-effectiveness in colorectal cancer screening is an important factor for policy decision making. This explains why costs and utilities are displayed as data items in the evaluation tool. However, for the version **1.0.0.**, information on costs and utilities are considered not relevant and no simulation results will be provided for cost-effectiveness of screening. Thus, costs in these quality checks are fixed and utilities may be imported using data from an exemplary country. If you still encounter problems with data quality, please contact us at: [eu.topia@erasmusmc.nl](mailto:eu.topia@erasmusmc.nl). Press “Continue” (**Supplementary** **Figure 6**).


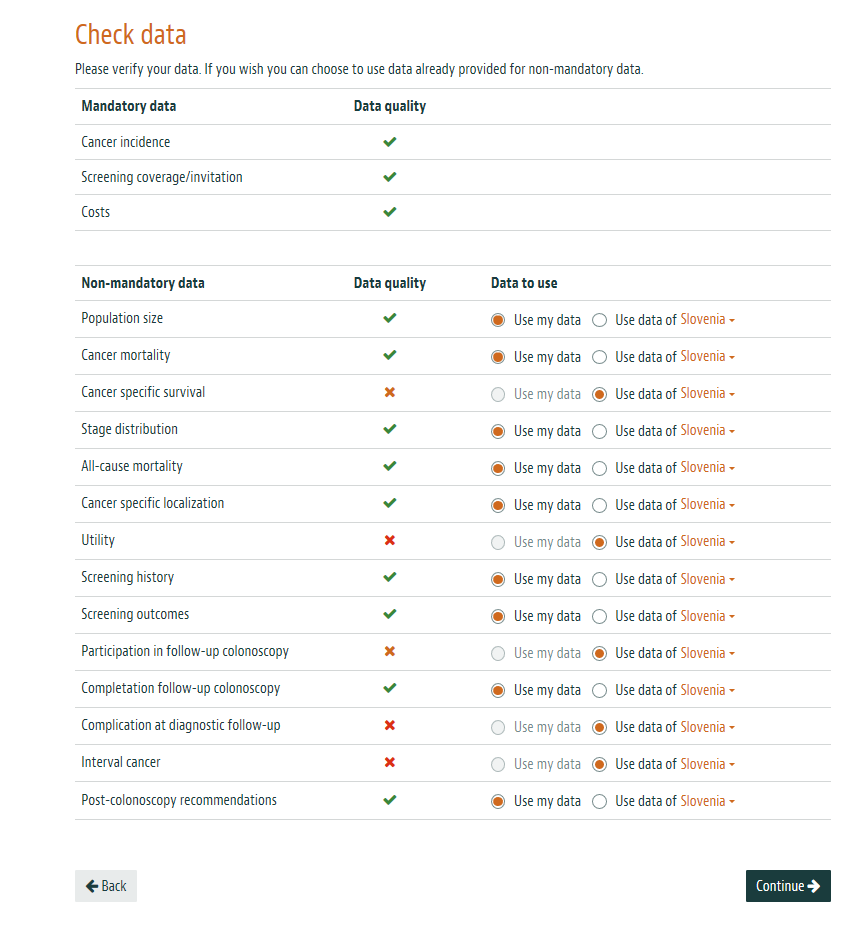


**Supplementary Figure 6**. EU-TOPIA evaluation tool, verify your data.

Selection of screening scenarios

After checking the quality of the data uploaded, your data are automatically converted in model parameters (please see next section for more details). Then, the evaluation tool will ask you to define the settings of each scenarios you want to simulate (**Supplementary Figure 7**). You can add more scenarios (a maximum of 5) by pressing the + sign. The default setting is the current status of your screening programme according to the mandatory data you provided (stratified by age, if provided with that option). You are able to change:

**Screening test:** Select one of the screening tests you want to use in this simulation. You may select to simulate screening with gFOBT, FIT (sensitivity and specificity adjusted with provided data for screening outcomes), Flexible-Sigmoidosocpy, Colonoscopy, FIT 20µg/gr (sensitivity and specificity calibrated using data from Imperiale et al. 2014, NEJM), and FIT 47µg/gr (sensitivity and specificity calibrated using data from The Netherlands Colorectal Cancer Screening programme).

**Target age**: Select the starting and stopping age at which individuals should be screened in this simulation. Please note that screening cannot be stratified by age in this phase of the simulation (age stratified screening may be incorporated in the model only as provided in the colorectal excel data template).

**Screening interval:** Select how many years there should be between each screening round.

**Adherence:** Select how much the participation rates should be reduced or increased. Please note that those values are in percentage points, so for example, assuming an adherence rates of 71%, selecting -25%, the new model adherence will be 46%.

**Invitation:** Select how much the invitation rates should be increased. Please note that those values are in percentage points, so for example, assuming an invitation rate of 25%, selecting +50%, the new model invitation will be 75%.

Please note these important remarks:

1. All the scenarios provided will be simulated after 2018 (including changes in coverage), before that year the model will assume the screening strategy provided in the data uploaded.
2. Current screening is the screening strategy provided in the uploaded data. It has to be thought as a block (Test, Age target, and Interval) and the evaluation tool does not allow to split this block. If you split the block, such as Test = Current, Age Target= Current, and Interval=5 years, you will not be allowed to run the simulation. However, you can change the coverage option in the current screening.

Press “Start simulation” (**Supplementary Figure 7**). Confirm that you are sure you want to start your simulation. Your simulation will be submitted for processing. The EU-TOPIA admins will notify you by e-mail as soon as your simulation is finished.


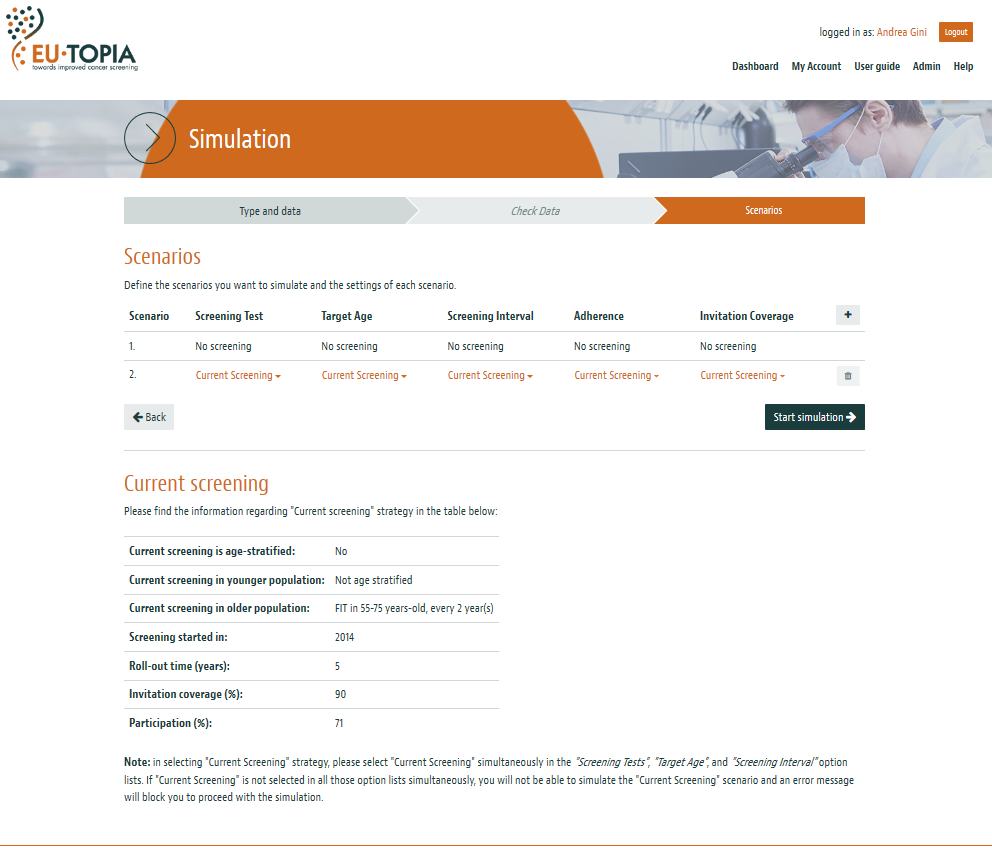


**Supplementary Figure 7**. EU-TOPIA evaluation tool, choose scenarios and start simulation

**Results**

Once your simulation is finished and the results are ready, you will receive an e-mail with a link (**Supplementary Figure 8**). Following the link, you will reach your simulation online and be able to download a PDF report with the results (**Supplementary Figure 9** and **Supplementary Figure 10**)**.** Alternatively, you can also find the results by going to the results section available in the MISCAN web-based tool dashboard. You will reach the results of the simulation by clicking on the simulation (if you have not performed a simulation yet, this page will be empty). On the page with the results of your simulation, you can find a summary of your simulated screening scenarios and a summary of the results (reported in millions; for ages 40-100; and for the period 2015-2050). You can retrieve the PDF of the simulation report, including more detailed results by year, by pressing the button “download results”.


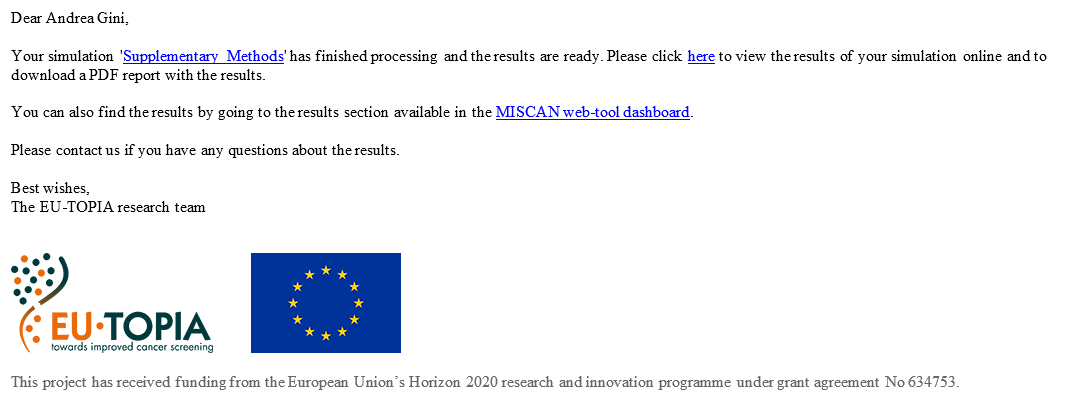


**Supplementary Figure 8**. EU-TOPIA evaluation tool, confirmation e-mail after simulation has finished processing.


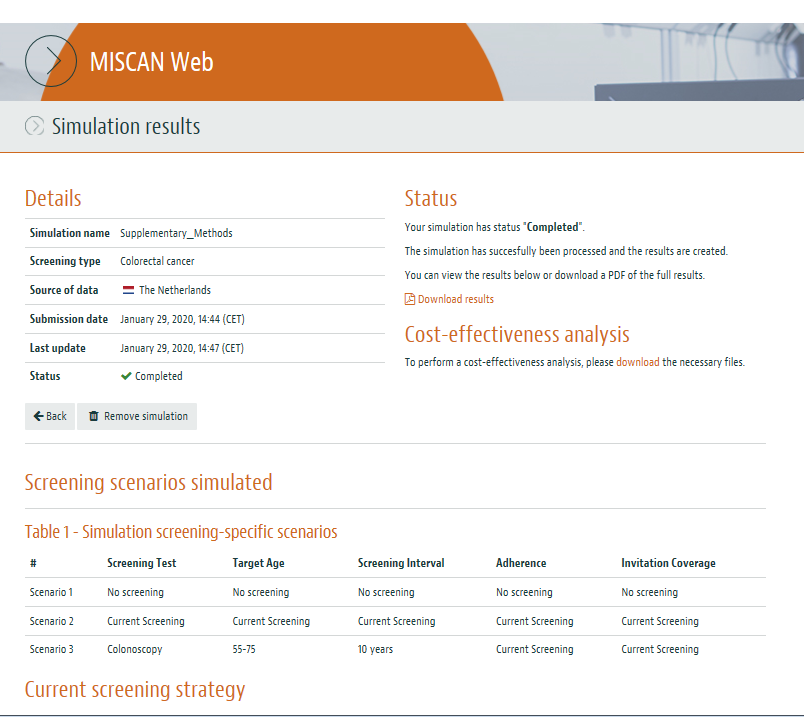


**Supplementary Figure 9**. EU-TOPIA evaluation tool, result web-page.


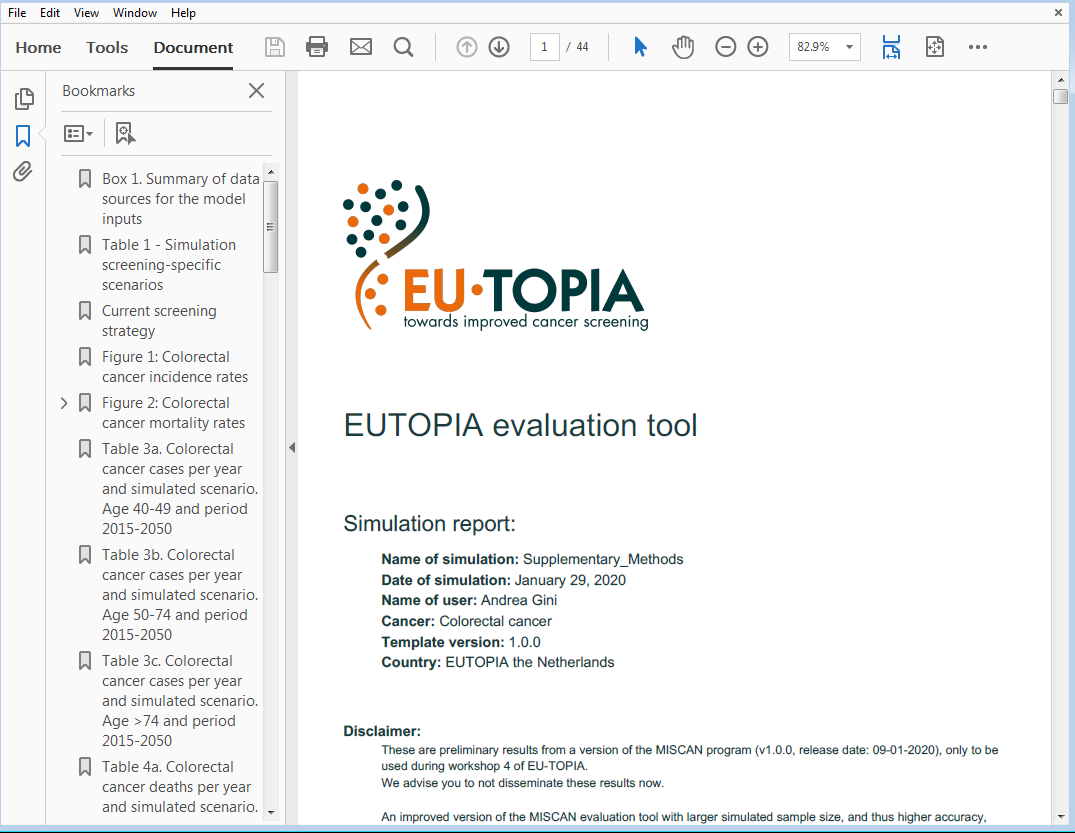


**Supplementary Figure 10**. EU-TOPIA evaluation tool, PDF report downloadable from the result web-page.
