# Supplementary material for: The EU-TOPIA evaluation tool: An online modelling-based tool for informing breast, cervical, and colorectal cancer screening decisions in Europe
Source: Prev Med Rep. 2021 Apr 30;22:101392. doi: 10.1016/j.pmedr.2021.101392 (PMC8122113; doi:10.1016/j.pmedr.2021.101392)
Supplement: Supplementary data 3 [file mmc3.pdf]

EU-TOPIA

Evaluation Tool

CANCER FACT SHEET: COLORECTAL CANCER (C18–C20)

MAY 2019

EU·TOPIA
towards improved cancer screening

SLOVENIA EXEMPLARY COUNTRY EAST

EU-TOPIA Evaluation Tool Cancer Fact Sheets: COLORECTAL CANCER (C18–C20)

FIGURE 1
Colorectal cancer age-specific incidence and mortality rates. Slovenia, 2004-2008.

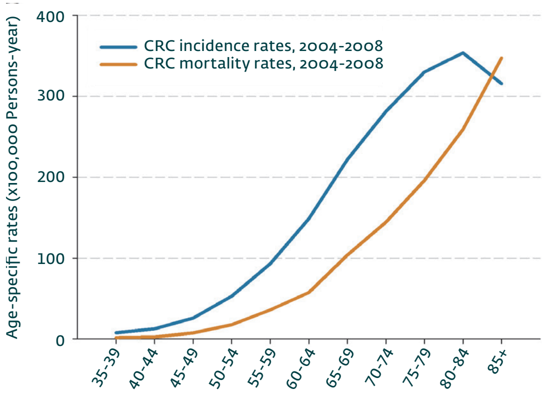

Notes
CRC, colorectal cancer; Slovenian Cancer Registry, 2004-2008.

FIGURE 3
Distribution of colorectal cancer localization. Slovenia, 2004-2008.

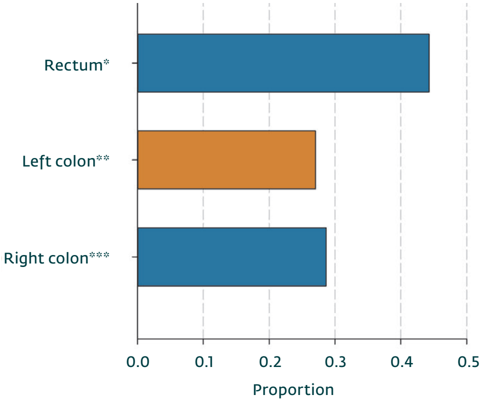

Source
Cancer Registry of Slovenia, 2004-2008;
** including rectumsigmoid;*
*** sigmoid and descending colon;*
**** transverse, flexures, ascending colon, and caecum.*

FIGURE 2
Colorectal cancer stage distribution in the pre-screening period. Slovenia, 2004-2008.

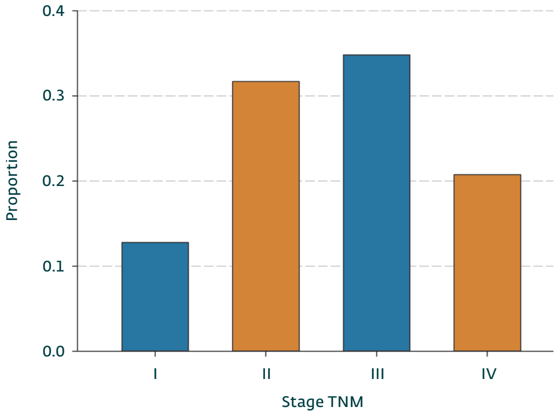

Source
Cancer Registry of Slovenia, 2004-2008.

FIGURE 4
Colorectal cancer 5-year relative survival. Slovenia, 2010-2014.

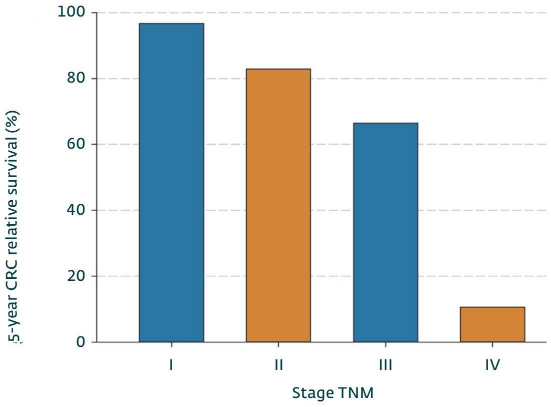

Source
Cancer Registry of Slovenia, 2010-2014.

Screening programme

Colorectal cancer (CRC) screening in Slovenia started in 2009, offering biennial fecal immunochemical test (FIT, 2-samples, at least one sample with a positivity cut-off of 100ng Hb/ml) among individuals aged 50-69 years. In the first round of the screening programme (2009-2011), adherence rate was 56.9% (303,343 participants) and FIT was positive in 6.2% of the participants. A total of 13,919 unsedated colonoscopies were performed with the ceacal intubation rate of 97.8%. The overall adenoma detection rate was 51.3%. Adenoma, advanced adenoma, or cancer were found in 7732 (55.5%) colonoscopies. A total of 862 (6.2%) CRC cases were found. In the second screening round (2011-2012) adherence rate was 57.8% and 493 CRC cases were found. From fourth screening round the eligible age is extended to 74 years of age.
Sources: Tepes et al. J Clin Gastroenterol 2016, Novak Mlakar et al. Radiol Oncol 2018, https://program-svit.si/en

FINLAND EXEMPLARY COUNTRY NORTH

EU-TOPIA Evaluation Tool Cancer Fact Sheets: COLORECTAL CANCER (C18–C20)

FIGURE 1
Colorectal cancer age-specific incidence and mortality rates.

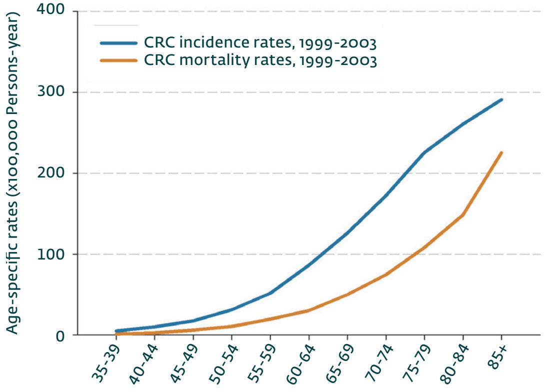

Notes
CRC, colorectal cancer; Source: Computed by the Erasmus team from Finnish Cancer Registry data, 1999-2003.

FIGURE 2
Colorectal cancer stage distribution in the pre-screening period.

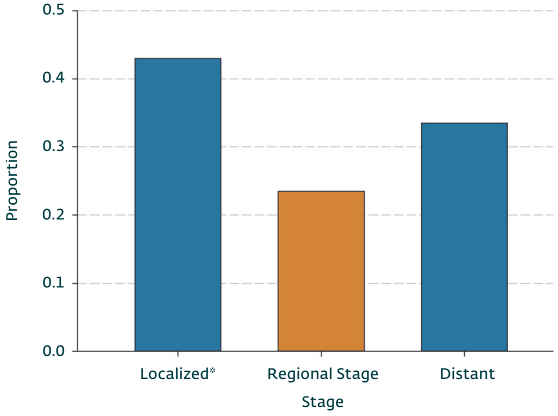

Source
Modified by the Erasmus team from Finnish Cancer Registry data, 1999-2003;
** one third assumed stage TNM I and two thirds assumed stage II;*
Regional assumed stage III; and Distant assumed stage IV (Finnish CRC staging data was not available according to the UICC TNM staging).

FIGURE 3
Distribution of colorectal cancer localization.

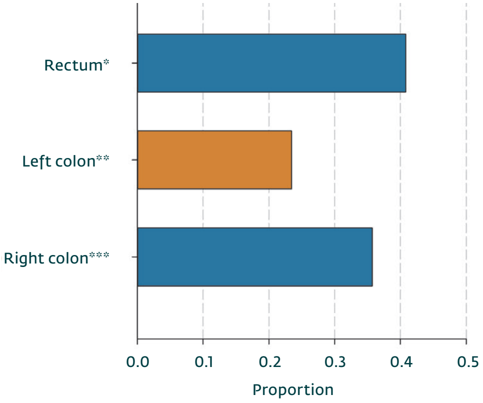

Source
Computed by the Erasmus team from Finnish Cancer Registry data, 1999-2003;
** including rectumsigmoid;*
*** sigmoid and descending colon;*
**** transverse, flexures, ascending colon, and caecum.*

FIGURE 4
Colorectal cancer gender-specific (Finland, 2010-2012) and model modified stage TNM-specific 5-year relative survivals.

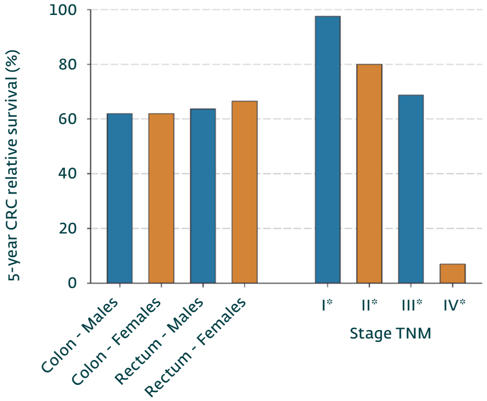

Source
Gender specific 5-year relative survival: Finnish Cancer Registry data (2010-2012);
**Stage TNM-specific 5 years relative survival were modified and adjusted by the Erasmus team from Finnish Cancer Registry data (2010-2012) comparing overall Finnish and Dutch CRC 5-year relative survival (Finnish CRC staging data was not available according to the UICC TNM staging).*

Screening programme
In Finland, a pilot study was carried out for testing the efficacy of biennial gFOBT screening among individuals aged 60-69 years. That large randomized study was gradually nested as part of the routine health services from 2004. A total of 180,210 subjects were included in the screening arm and 180,282 in the control arm.

In 2012, the screening programme covered 43% of the target age population in Finland. Uptake was 68.8% and the proportion of positive gFOBT was 3.6%. The proportion of screen-detected CRCs was 42.7% of all CRCs. Colonoscopy was performed in 84% of screen positives.'
Source: Pitkäniemi et al. BMJ Open Gastroenterology 2015.

ITALY EXEMPLARY COUNTRY SOUTH

EU-TOPIA Evaluation Tool Cancer Fact Sheets: COLORECTAL CANCER (C18–C20)

FIGURE 1

Colorectal cancer age-specific incidence and mortality rates. Italy, 1998-2002.

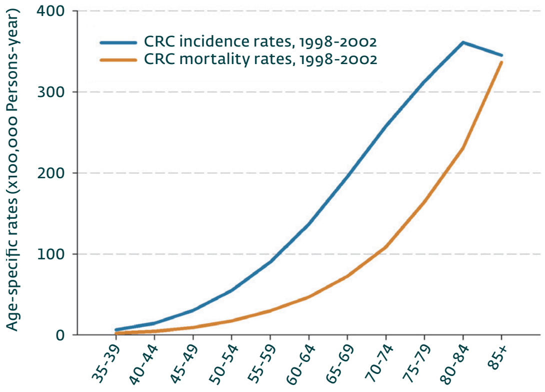

Notes
CRC, colorectal cancer;
Source incidence, IACR Cancer incidence in 5 continents (CI5-IX, more details in Figure 5 and Table 1;
Source mortality, ISS/ISTAT (https://www.iss.it/site/mortalita/Scripts/SelCause.asp. Last access: February 22, 2017).

FIGURE 3

Distribution of colorectal cancer localization. Italy, 1998-2002.

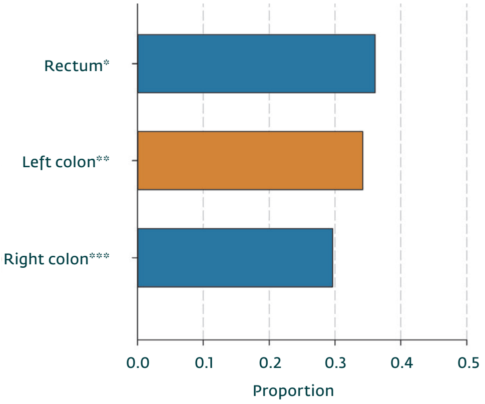

Source
IACR Cancer incidence in 5 continents (CI5-IX, more details in Figure 5 and Table 1);
** including rectumsigmoid;*
*** sigmoid and descending colon;*
**** transverse, flexures, ascending colon, and caecum.*

FIGURE 2

Colorectal cancer stage distribution in the pre-screening period.

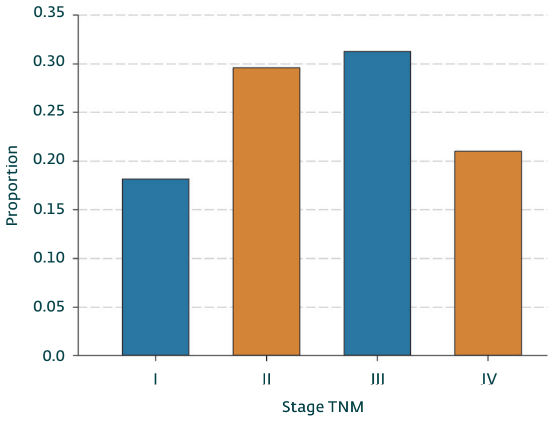

Source
Zorzi M (2015). Epid. Prev. 115-25.

FIGURE 4

Colorectal cancer 5-year relative survival. Italy, 2003-2005.

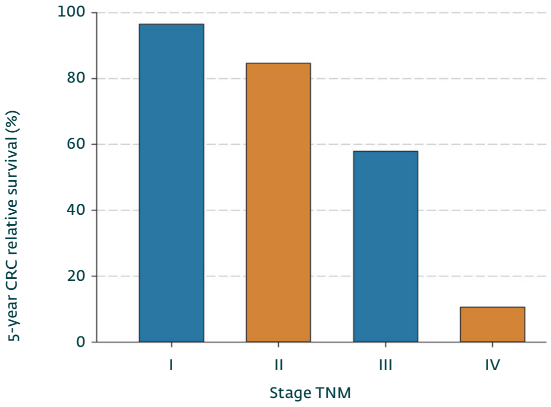

Source
Minicozzi P, et al. European Journal of Public Health, 24, No. 1, 98-100..

EU·TOPIA
towards improved cancer screening

FIGURE 5
Cancer registry data included.
Italy, 1998-2002

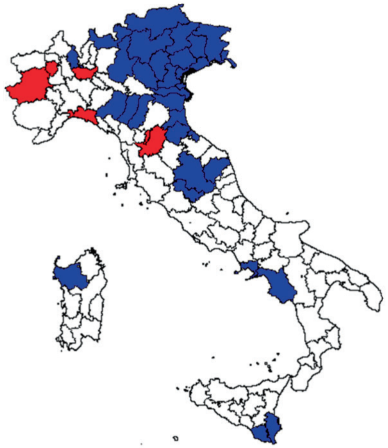

CRs Included: No screening
CRS Excluded: Pilot/PB screening

Notes
Romagna Region included individuals aged 55-64 invited in SCORE trials (0.6%); Veneto Region provided to IARC data in period 1998-2001 and started FIT screening in May 2002; CRC, colorectal cancer; CRs, Cancer Registries; IARC Cancer Incidence in 5 Continents (CI5-IX).

Notes table 1 →
Marked CRs presented in Table 1 were excluded in the CRC incidence rates due to early or pilot implementation of CRC screening.

TABLE 1
Cancer registry data included.
Italy, 1998-2002

ITALIAN CRS, 1998-2002
Biella Province (1998-2002)
Brescia Province (1999-2001)
Ferrara Province (1998-2002)
Florence and Prato (1998-2002)
Genoa Province (1998-2000)
Macerata Province (1998-2000)
Milan (1999-2002)
Modena Province (1998-2002)
Naples (1998-2002)
North East Cancer Surveillance Network (1998-2002)
Parma Province (1998-2002)
Ragusa Province (1998-2002)
Reggio Emilia Province (1998-2002)
Romagna Region (1998-2002)
Salerno Province (1998-2001)
Sassari Province (1998-2002)
Syracuse Province (1999-2002)
Sondrio (1998-2002)
Turin (1998-2002)
Umbria Region (1998-2002)
Varese Province (1998-2000)
Veneto Region (1998-2001)

Screening programme
'Colorectal cancer (CRC) screening in Italy started in the early 2000, but it was not implemented homogenously across the country. By the end of 2016, 117 local programs were active. The majority of programs offer biennial fecal immunochemical test (FIT, positivity cut-off 20 µg Hb/gr.faeces), while some adopted flexible sigmoidoscopy (FS) once in a lifetime and FIT for nonresponders to FS. The national invitation coverage of the target 50-69 population was 76.4 (1). Screening performance is monitored on a yearly basis and data about the main quality indicators of the screening process are available at www.osservatorionazionalescreening.it. The most recent published report (2) documented CRC screening activity in 2011-12.

FIT screening
During the 2-year interval 2011-12, 7,744,000 subjects were invited to undergo FIT: adjusted attendance rate was 47.1% and 3,531,937 subjects were screened. Positivity rate of FIT programs was 5.2% at first screening and 4.0% at repeat screening.
The average attendance rate to total colonoscopy (TC) was 81.2%. Completion rate for TC was 91%. The detection rate (DR) per 1,000 screened subjects was 2.0 for invasive cancer and 9.1‰ for advanced adenomas (AA, adenomas with a diameter ≥1 cm, with villous/tubulo-villous type or high-grade dysplasia) at first and 1.0 for CRC and 6.8 for AA at repeat screening.

FS screening
During the 2-year interval 2011-12, 24,549 subjects were screened in the two years, with an attendance rate of 24.5%. The programmes offer FIT to subjects refusing FS screening. This strategy makes it possible to increase overall coverage: in fact, the proportion of subjects who underwent one of the two tests was 36.4%. Overall, 85.9% of FSs were classified as complete. Overall, TC referral rate was 9.8% and the DR per 1,000 screened subjects was 3.0 and 48.2 for invasive cancer and AA, respectively.'

Source:
1) www.osservatorionazionalescreening.it/ sites/default/files/allegati/ons%20rapporto%202017.pdf
2) Zorzi et al. Epidemiol Prev 2015; 39(3) Suppl 1: 93-107

EU·TOPIA
towards improved cancer screening

THE NETHERLANDS EXEMPLARY COUNTRY WEST

EEU-TOPIA Evaluation Tool Cancer Fact Sheets: COLORECTAL CANCER (C18–C20)

FIGURE 1

Colorectal cancer age-specific incidence and mortality rates. The Netherlands, 2009-2013.

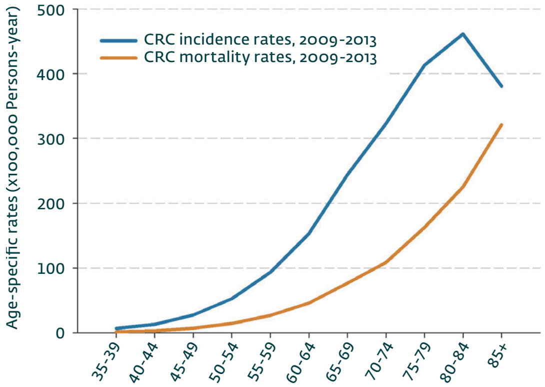

Notes

CRC, colorectal cancer; Dutch Cancer Registry, 2009-2013.

FIGURE 2

Colorectal cancer stage distribution in the pre-screening period. The Netherlands, 2009-2013.

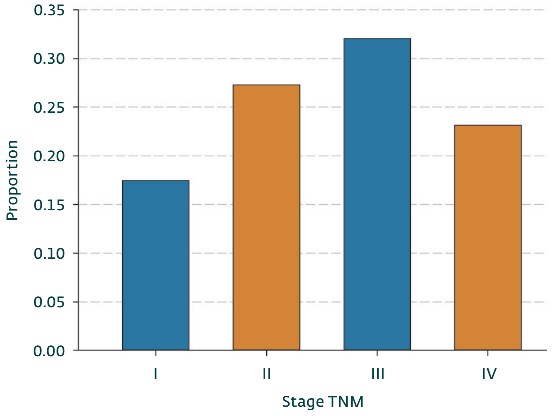

Source

Dutch Cancer Registry, 2009-2013.

FIGURE 3

Distribution of colorectal cancer localization. The Netherlands, 2009-2013.

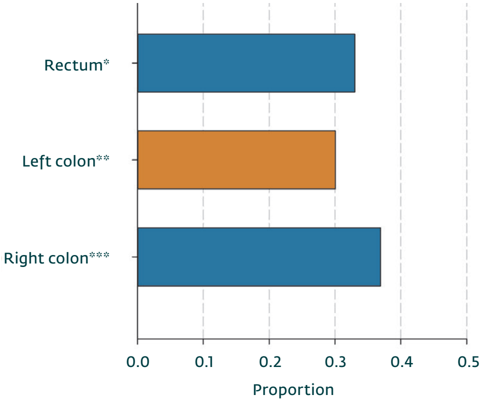

Source

Dutch Cancer Registry, 2009-2013;

* *including rectumsigmoid;*

** *sigmoid and descending colon;*

*** *transverse, flexures, ascending colon, and caecum.*

FIGURE 4

Colorectal cancer 5-year relative survival. The Netherlands, 2009-2013.

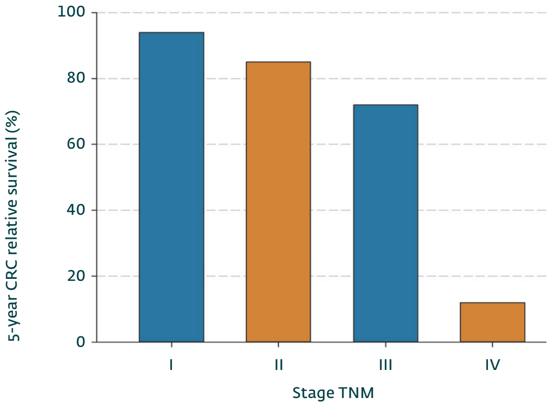

Source

Dutch Cancer Registry, 2009-2013.

Screening programme

The Dutch national screening program for colorectal cancer (CRC), with biennial fecal immunochemical tests (FITs), was initiated in 2014. Among 741,914 persons invited for FIT, 529,056 (71.3%) participated. Positive test results (10.6%) were higher than predicted based on pilot studies and the PPV was lower (42.1%). Detection rates of CRC and AA were 5.8% and 30.8%, respectively. Cut-off level for a positive FIT result was increased from 15 to 47 µg Hb/g feces halfway through 2014, to reduce the burden of unnecessary colonoscopies. Positive test results decreased to 6.7% and PPV increased up to 49.1%.'

Source: Toes-Zoutendijk et al. Gastroenterology 2017.

EU·TOPIA
towards improved cancer screening
