# Supplementary material for: The EU-TOPIA evaluation tool: An online modelling-based tool for informing breast, cervical, and colorectal cancer screening decisions in Europe
Source: Prev Med Rep. 2021 Apr 30;22:101392. doi: 10.1016/j.pmedr.2021.101392 (PMC8122113; doi:10.1016/j.pmedr.2021.101392)
Supplement: Supplementary data 4 [file mmc4.pdf]

EUTOPIA evaluation tool

Simulation report:

Name of simulation: Supplementary_Methods
Date of simulation: January 29, 2020
Name of user: Andrea Gini
Cancer: Colorectal cancer
Template version: 1.0.0
Country: EUTOPIA the Netherlands

Disclaimer:

These are preliminary results from a version of the MISCAN program (v1.0.0, release date: 09-01-2020), only to be used during workshop 4 of EU-TOPIA.
We advise you to not disseminate these results now.

An improved version of the MISCAN evaluation tool with larger simulated sample size, and thus higher accuracy, will be available after the workshop.
Neither the EU-TOPIA consortium nor the Department of Public Health (Erasmus MC University Medical Center Rotterdam) can be held liable in any way for any results of the program.

These results or any portion thereof may not be reproduced or used in any manner whatsoever without the express written permission of
Prof. dr. Harry de Koning, MD
(P.I. EU-TOPIA project)
Erasmus MC
University Medical Center Rotterdam
Department of Public Health

towards improved cancer screening

Box 1. Summary of data sources for the model inputs

Data	User's selection
Cancer incidence	EUTOPIA the Netherlands
Screening coverage/invitation	EUTOPIA the Netherlands
Country	EUTOPIA the Netherlands
Population size	EUTOPIA the Netherlands
Cancer mortality	EUTOPIA the Netherlands
Cancer specific survival	EUTOPIA the Netherlands
Stage distribution	EUTOPIA the Netherlands
All-cause mortality	EUTOPIA the Netherlands
Cancer specific localization	EUTOPIA the Netherlands
Utility	EUTOPIA the Netherlands
Screening history	EUTOPIA the Netherlands
Screening outcomes	EUTOPIA the Netherlands
Participation in follow-up colonoscopy	EUTOPIA the Netherlands
Completation follow-up colonoscopy	EUTOPIA the Netherlands
Complication at diagnostic follow-up	EUTOPIA the Netherlands
Interval cancer	EUTOPIA the Netherlands
Post-colonoscopy recommendations	EUTOPIA the Netherlands

Screening scenarios simulated

Table 1 - Simulation screening-specific scenarios

#	Screening Test	Target Age	Screening Interval	Adherence	Invitation Coverage
Scenario 1	No screening	No screening	No screening	No screening	No screening
Scenario 2	Current Screening	Current Screening	Current Screening	Current Screening	Current Screening
Scenario 3	Colonoscopy	55-75	10 years	Current Screening	Current Screening

Current screening strategy

**Current screening is age-stratified:**	No
**Current screening in younger population:**	Not age stratified
**Current screening in older population:**	FIT in 55-75 years-old, every 2 year(s)
**Screening started in:**	2014
**Roll-out time (years):**	5
**Invitation coverage (%):**	90
**Participation (%):**	71

Figure 1: Colorectal cancer incidence rates

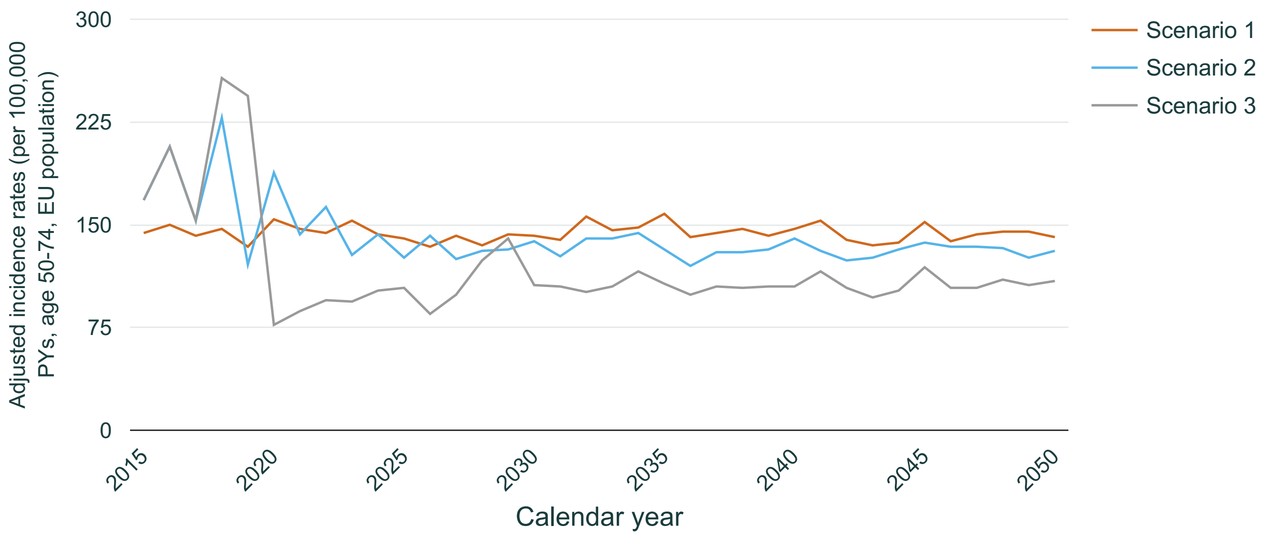

Figure 2: Colorectal cancer mortality rates

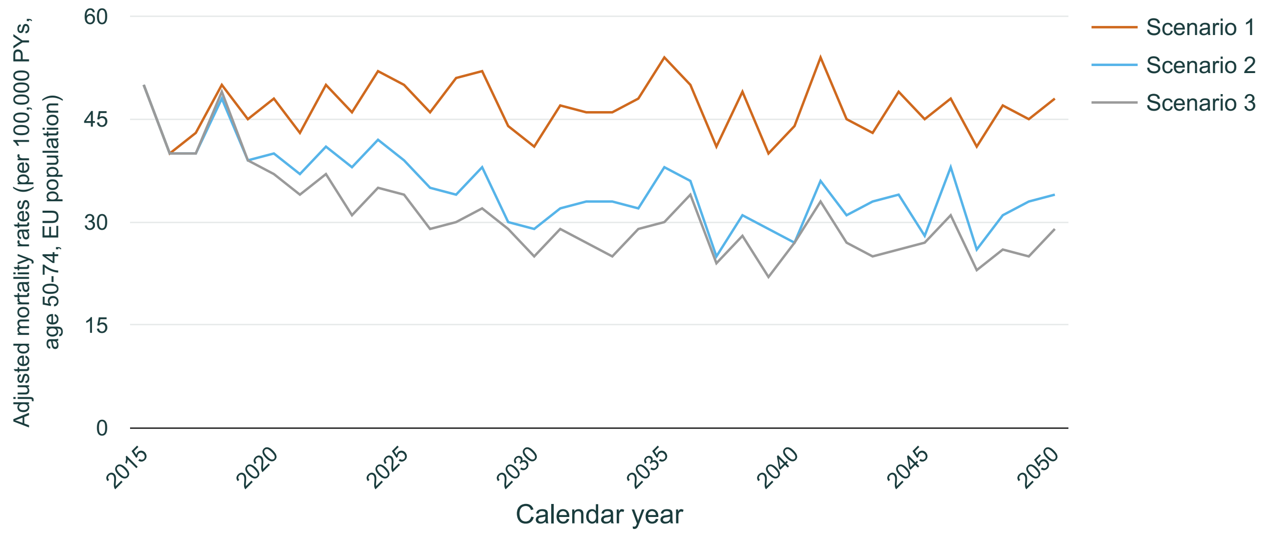

Table 2. Screening outcomes (x10,000) for each simulated scenario in individuals aged 40-100, 2018-2050

	Scenario 1	Scenario 2	Scenario 3
Population older than 40 years in 2018	912.33	912.33	912.33
Person-years, 2018-2050	33118.9	33118.9	33118.9
Colorectal cancer cases (age 40-100)	56.49	48.27	40.11
Colorectal cancer incidence reduction (%,40-100) compared to no screening	0	14.56	29
Colorectal cancer deaths (age 40-100)	24.18	17.8	15.37
Colorectal cancer mortality reduction (%,40-100) compared to no screening	0	26.39	36.44
Colorectal cancer cases (age 50-74)	27.02	25.61	20.94
Colorectal cancer incidence reduction (%,50-74) compared to no screening	0	5.23	22.5
Colorectal cancer deaths (age 50-74)	8.87	6.39	5.58
Colorectal cancer mortality reduction (%,50-74) compared to no screening	0	27.92	37.1
Primary screening tests:			
- gFOBT	0	0	0
- FIT	0	4731.24	0
- FS	0	0	0
- Colonoscopy	0	0	1242.13
Positive screening tests	0	192.87	402.58
Diagnostic follow-up colonoscopies performed	0	164.01	0
False Positive (%)	0	2.27	0
Colonoscopy complications	0	1.85	5.88
Adenomas Detected	0	88.59	260.26
Colorectal Cancers Detected	0	9.8	5.65

Table 3a. Colorectal cancer cases per year and simulated scenario. Age 40-49 and period 2015-2050

Year	Scenario 1	Scenario 2	Scenario 3
2015	627	627	627
2016	381	381	381
2017	344	344	344
2018	345	345	345
2019	153	153	153
2020	402	402	402
2021	476	476	476
2022	347	347	347
2023	395	395	395
2024	317	317	317
2025	375	375	375
2026	584	584	584
2027	392	392	392
2028	335	335	335
2029	637	637	637
2030	454	454	454
2031	398	398	398
2032	513	513	513
2033	341	341	341
2034	370	370	370
2035	397	397	397
2036	376	376	376
2037	332	332	332
2038	523	523	523
2039	382	382	382
2040	536	536	536
2041	564	564	564
2042	347	347	347
2043	430	430	430
2044	400	400	400
2045	330	330	330
2046	382	382	382
2047	540	540	540
2048	382	382	382
2049	569	569	569
2050	254	254	254

Table 3b. Colorectal cancer cases per year and simulated scenario. Age 50-74 and period 2015-2050

Year	Scenario 1	Scenario 2	Scenario 3
2015	7343	8657	8657
2016	7826	10667	10667
2017	7503	8090	8090
2018	7914	12270	13826
2019	7358	6671	13448
2020	8578	10524	4300
2021	8356	8143	4966
2022	8181	9249	5412
2023	8692	7280	5341
2024	8178	8179	5806
2025	8021	7226	5976
2026	7774	8229	4893
2027	8292	7281	5730
2028	7911	7641	7250
2029	8439	7779	8270
2030	8435	8129	6235
2031	8348	7507	6191
2032	9280	8189	5801
2033	8550	8009	5959
2034	8652	8323	6629
2035	9330	7643	6173
2036	8427	6962	5726
2037	8439	7473	5968
2038	8492	7389	5883
2039	8204	7402	5859
2040	8271	7830	5792
2041	8631	7258	6471
2042	7786	6817	5741
2043	7589	6897	5326
2044	7613	7238	5594
2045	8348	7407	6460
2046	7459	7150	5568
2047	7633	7148	5498
2048	7698	7055	5823
2049	7741	6728	5643
2050	7576	7041	5840

EU·TOPIA

Table 3c. Colorectal cancer cases per year and simulated scenario. Age >74 and period 2015-2050

Year	Scenario 1	Scenario 2	Scenario 3
2015	5040	5040	5040
2016	4520	4520	4520
2017	4894	4894	4894
2018	5398	5398	5398
2019	5954	5874	5874
2020	5355	5548	4870
2021	5212	4747	4543
2022	6059	5393	5041
2023	6433	5431	4900
2024	6617	5794	5247
2025	7129	5530	4721
2026	6789	5135	4431
2027	7541	5758	4888
2028	6262	4962	3995
2029	7882	6005	5192
2030	7770	5654	4877
2031	7776	5601	4836
2032	8080	6176	5010
2033	8732	6260	5324
2034	8226	5554	4463
2035	9451	6973	5675
2036	9619	7444	5369
2037	10003	7382	5778
2038	9814	6829	5885
2039	9888	6998	5965
2040	10272	7270	6032
2041	9687	7028	5958
2042	10732	7430	6324
2043	10217	7924	6509
2044	10273	7174	5893
2045	10494	7542	5913
2046	10405	7246	5083
2047	10450	7894	6466
2048	10693	7498	5857
2049	10961	7776	5974
2050	10983	7810	5834

EU·TOPIA

Table 4a. Colorectal cancer deaths per year and simulated scenario. Age 40-49 and period 2015-2050

Year	Scenario 1	Scenario 2	Scenario 3
2015	113	113	113
2016	95	95	95
2017	134	134	134
2018	96	96	96
2019	96	96	96
2020	39	39	39
2021	78	78	78
2022	176	176	176
2023	179	179	179
2024	78	78	78
2025	102	102	102
2026	79	79	79
2027	62	62	62
2028	210	210	210
2029	85	85	85
2030	111	111	111
2031	108	108	108
2032	67	67	67
2033	45	45	45
2034	161	161	161
2035	47	47	47
2036	209	209	209
2037	71	71	71
2038	168	168	168
2039	25	25	25
2040	244	244	244
2041	150	150	150
2042	123	123	123
2043	127	127	127
2044	77	77	77
2045	254	254	254
2046	128	128	128
2047	26	26	26
2048	230	230	230
2049	53	53	53
2050	256	256	256

Table 4b. Colorectal cancer deaths per year and simulated scenario. Age 50-74 and period 2015-2050

Year	Scenario 1	Scenario 2	Scenario 3
2015	2498	2498	2498
2016	2041	2001	2001
2017	2293	2141	2141
2018	2701	2603	2623
2019	2500	2145	2146
2020	2673	2242	2042
2021	2477	2105	1945
2022	2822	2303	2086
2023	2582	2150	1758
2024	2937	2391	2002
2025	2867	2203	1933
2026	2699	2024	1694
2027	2940	1986	1709
2028	3082	2215	1880
2029	2593	1794	1697
2030	2457	1689	1469
2031	2786	1877	1694
2032	2792	1922	1601
2033	2691	1868	1414
2034	2850	1803	1666
2035	3265	2275	1732
2036	3013	2181	2022
2037	2459	1443	1366
2038	2893	1754	1571
2039	2377	1648	1283
2040	2630	1582	1579
2041	3101	2063	1855
2042	2553	1762	1504
2043	2403	1806	1392
2044	2694	1865	1408
2045	2504	1571	1488
2046	2619	2033	1677
2047	2218	1369	1242
2048	2501	1668	1401
2049	2416	1763	1352
2050	2575	1810	1540

Table 4c. Colorectal cancer deaths per year and simulated scenario. Age >74 and period 2015-2050

Year	Scenario 1	Scenario 2	Scenario 3
2015	2325	2325	2325
2016	2737	2737	2737
2017	2665	2665	2665
2018	2629	2629	2629
2019	2528	2508	2508
2020	3002	2924	2924
2021	2779	2667	2630
2022	2991	2853	2775
2023	3210	3066	2882
2024	3179	2799	2736
2025	3897	3225	2886
2026	4062	3243	3060
2027	3768	3014	2690
2028	3516	2777	2419
2029	3670	2912	2596
2030	4321	3193	2746
2031	3957	3182	2593
2032	4242	3211	2636
2033	4346	3258	2599
2034	4670	3334	2905
2035	4350	3224	2654
2036	4656	3007	2635
2037	5597	4018	3288
2038	5443	3834	3113
2039	5582	3839	3177
2040	5429	3623	2760
2041	5397	3364	2640
2042	5442	3655	2936
2043	5242	3512	3046
2044	5273	3434	3056
2045	5511	3616	3163
2046	5197	3375	2344
2047	6535	4456	3629
2048	5999	3898	2907
2049	6586	4488	3203
2050	6118	3956	3166

Table 5a. Screening gFOBT per year and simulated scenario. Age 40-49 and period 2015-2050

Year	Scenario 1	Scenario 2	Scenario 3
2015	0	0	0
2016	0	0	0
2017	0	0	0
2018	0	0	0
2019	0	0	0
2020	0	0	0
2021	0	0	0
2022	0	0	0
2023	0	0	0
2024	0	0	0
2025	0	0	0
2026	0	0	0
2027	0	0	0
2028	0	0	0
2029	0	0	0
2030	0	0	0
2031	0	0	0
2032	0	0	0
2033	0	0	0
2034	0	0	0
2035	0	0	0
2036	0	0	0
2037	0	0	0
2038	0	0	0
2039	0	0	0
2040	0	0	0
2041	0	0	0
2042	0	0	0
2043	0	0	0
2044	0	0	0
2045	0	0	0
2046	0	0	0
2047	0	0	0
2048	0	0	0
2049	0	0	0
2050	0	0	0

EU·TOPIA

Table 5b. Screening gFOBT per year and simulated scenario. Age 50-74 and period 2015-2050

Year	Scenario 1	Scenario 2	Scenario 3
2015	0	0	0
2016	0	0	0
2017	0	0	0
2018	0	0	0
2019	0	0	0
2020	0	0	0
2021	0	0	0
2022	0	0	0
2023	0	0	0
2024	0	0	0
2025	0	0	0
2026	0	0	0
2027	0	0	0
2028	0	0	0
2029	0	0	0
2030	0	0	0
2031	0	0	0
2032	0	0	0
2033	0	0	0
2034	0	0	0
2035	0	0	0
2036	0	0	0
2037	0	0	0
2038	0	0	0
2039	0	0	0
2040	0	0	0
2041	0	0	0
2042	0	0	0
2043	0	0	0
2044	0	0	0
2045	0	0	0
2046	0	0	0
2047	0	0	0
2048	0	0	0
2049	0	0	0
2050	0	0	0

EU·TOPIA

Table 5c. Screening gFOBT per year and simulated scenario. Age >74 and period 2015-2050

Year	Scenario 1	Scenario 2	Scenario 3
2015	0	0	0
2016	0	0	0
2017	0	0	0
2018	0	0	0
2019	0	0	0
2020	0	0	0
2021	0	0	0
2022	0	0	0
2023	0	0	0
2024	0	0	0
2025	0	0	0
2026	0	0	0
2027	0	0	0
2028	0	0	0
2029	0	0	0
2030	0	0	0
2031	0	0	0
2032	0	0	0
2033	0	0	0
2034	0	0	0
2035	0	0	0
2036	0	0	0
2037	0	0	0
2038	0	0	0
2039	0	0	0
2040	0	0	0
2041	0	0	0
2042	0	0	0
2043	0	0	0
2044	0	0	0
2045	0	0	0
2046	0	0	0
2047	0	0	0
2048	0	0	0
2049	0	0	0
2050	0	0	0

Table 6a. Screening FIT per year and simulated scenario. Age 40-49 and period 2015-2050

Year	Scenario 1	Scenario 2	Scenario 3
2015	0	0	0
2016	0	0	0
2017	0	0	0
2018	0	0	0
2019	0	0	0
2020	0	0	0
2021	0	0	0
2022	0	0	0
2023	0	0	0
2024	0	0	0
2025	0	0	0
2026	0	0	0
2027	0	0	0
2028	0	0	0
2029	0	0	0
2030	0	0	0
2031	0	0	0
2032	0	0	0
2033	0	0	0
2034	0	0	0
2035	0	0	0
2036	0	0	0
2037	0	0	0
2038	0	0	0
2039	0	0	0
2040	0	0	0
2041	0	0	0
2042	0	0	0
2043	0	0	0
2044	0	0	0
2045	0	0	0
2046	0	0	0
2047	0	0	0
2048	0	0	0
2049	0	0	0
2050	0	0	0

Table 6b. Screening FIT per year and simulated scenario. Age 50-74 and period 2015-2050

Year	Scenario 1	Scenario 2	Scenario 3
2015	0	697337	697337
2016	0	1038024	1038024
2017	0	839871	839871
2018	0	1843146	0
2019	0	966099	0
2020	0	1747886	0
2021	0	1127973	0
2022	0	1739612	0
2023	0	1141575	0
2024	0	1614015	0
2025	0	1271869	0
2026	0	1622757	0
2027	0	1305652	0
2028	0	1513197	0
2029	0	1402499	0
2030	0	1498793	0
2031	0	1387828	0
2032	0	1364803	0
2033	0	1482990	0
2034	0	1351979	0
2035	0	1333595	0
2036	0	1323911	0
2037	0	1305472	0
2038	0	1298622	0
2039	0	1292797	0
2040	0	1264168	0
2041	0	1265160	0
2042	0	1245514	0
2043	0	1247240	0
2044	0	1252870	0
2045	0	1238888	0
2046	0	1245620	0
2047	0	1231984	0
2048	0	1251718	0
2049	0	1265177	0
2050	0	1273742	0

EU·TOPIA

Table 6c. Screening FIT per year and simulated scenario. Age >74 and period 2015-2050

Year	Scenario 1	Scenario 2	Scenario 3
2015	0	0	0
2016	0	0	0
2017	0	0	0
2018	0	0	0
2019	0	0	0
2020	0	105597	0
2021	0	0	0
2022	0	0	0
2023	0	0	0
2024	0	108081	0
2025	0	0	0
2026	0	0	0
2027	0	0	0
2028	0	128965	0
2029	0	0	0
2030	0	0	0
2031	0	0	0
2032	0	109970	0
2033	0	0	0
2034	0	0	0
2035	0	133317	0
2036	0	133018	0
2037	0	137765	0
2038	0	136547	0
2039	0	131560	0
2040	0	140150	0
2041	0	140964	0
2042	0	143104	0
2043	0	145106	0
2044	0	129672	0
2045	0	140068	0
2046	0	135573	0
2047	0	135830	0
2048	0	136515	0
2049	0	109839	0
2050	0	111624	0

EU·TOPIA

Table 7a. Screening FS per year and simulated scenario. Age 40-49 and period 2015-2050

Year	Scenario 1	Scenario 2	Scenario 3
2015	0	0	0
2016	0	0	0
2017	0	0	0
2018	0	0	0
2019	0	0	0
2020	0	0	0
2021	0	0	0
2022	0	0	0
2023	0	0	0
2024	0	0	0
2025	0	0	0
2026	0	0	0
2027	0	0	0
2028	0	0	0
2029	0	0	0
2030	0	0	0
2031	0	0	0
2032	0	0	0
2033	0	0	0
2034	0	0	0
2035	0	0	0
2036	0	0	0
2037	0	0	0
2038	0	0	0
2039	0	0	0
2040	0	0	0
2041	0	0	0
2042	0	0	0
2043	0	0	0
2044	0	0	0
2045	0	0	0
2046	0	0	0
2047	0	0	0
2048	0	0	0
2049	0	0	0
2050	0	0	0

Table 7b. Screening FS per year and simulated scenario. Age 50-74 and period 2015-2050

Year	Scenario 1	Scenario 2	Scenario 3
2015	0	0	0
2016	0	0	0
2017	0	0	0
2018	0	0	0
2019	0	0	0
2020	0	0	0
2021	0	0	0
2022	0	0	0
2023	0	0	0
2024	0	0	0
2025	0	0	0
2026	0	0	0
2027	0	0	0
2028	0	0	0
2029	0	0	0
2030	0	0	0
2031	0	0	0
2032	0	0	0
2033	0	0	0
2034	0	0	0
2035	0	0	0
2036	0	0	0
2037	0	0	0
2038	0	0	0
2039	0	0	0
2040	0	0	0
2041	0	0	0
2042	0	0	0
2043	0	0	0
2044	0	0	0
2045	0	0	0
2046	0	0	0
2047	0	0	0
2048	0	0	0
2049	0	0	0
2050	0	0	0

Table 7c. Screening FS per year and simulated scenario. Age >74 and period 2015-2050

Year	Scenario 1	Scenario 2	Scenario 3
2015	0	0	0
2016	0	0	0
2017	0	0	0
2018	0	0	0
2019	0	0	0
2020	0	0	0
2021	0	0	0
2022	0	0	0
2023	0	0	0
2024	0	0	0
2025	0	0	0
2026	0	0	0
2027	0	0	0
2028	0	0	0
2029	0	0	0
2030	0	0	0
2031	0	0	0
2032	0	0	0
2033	0	0	0
2034	0	0	0
2035	0	0	0
2036	0	0	0
2037	0	0	0
2038	0	0	0
2039	0	0	0
2040	0	0	0
2041	0	0	0
2042	0	0	0
2043	0	0	0
2044	0	0	0
2045	0	0	0
2046	0	0	0
2047	0	0	0
2048	0	0	0
2049	0	0	0
2050	0	0	0

Table 8a. Screening colonoscopies per year and simulated scenario. Age 40-49 and period 2015-2050

Year	Scenario 1	Scenario 2	Scenario 3
2015	0	0	0
2016	0	0	0
2017	0	0	0
2018	0	0	0
2019	0	0	0
2020	0	0	0
2021	0	0	0
2022	0	0	0
2023	0	0	0
2024	0	0	0
2025	0	0	0
2026	0	0	0
2027	0	0	0
2028	0	0	0
2029	0	0	0
2030	0	0	0
2031	0	0	0
2032	0	0	0
2033	0	0	0
2034	0	0	0
2035	0	0	0
2036	0	0	0
2037	0	0	0
2038	0	0	0
2039	0	0	0
2040	0	0	0
2041	0	0	0
2042	0	0	0
2043	0	0	0
2044	0	0	0
2045	0	0	0
2046	0	0	0
2047	0	0	0
2048	0	0	0
2049	0	0	0
2050	0	0	0

EU·TOPIA

Table 8b. Screening colonoscopies per year and simulated scenario. Age 50-74 and period 2015-2050

Year	Scenario 1	Scenario 2	Scenario 3
2015	0	0	0
2016	0	0	0
2017	0	0	0
2018	0	0	1156111
2019	0	0	1608109
2020	0	0	165735
2021	0	0	165888
2022	0	0	172813
2023	0	0	172556
2024	0	0	153630
2025	0	0	164035
2026	0	0	163225
2027	0	0	160002
2028	0	0	588486
2029	0	0	897731
2030	0	0	251432
2031	0	0	249513
2032	0	0	254522
2033	0	0	256622
2034	0	0	240841
2035	0	0	252739
2036	0	0	244597
2037	0	0	242504
2038	0	0	240810
2039	0	0	234304
2040	0	0	229246
2041	0	0	223834
2042	0	0	227759
2043	0	0	228850
2044	0	0	244500
2045	0	0	248005
2046	0	0	245373
2047	0	0	240788
2048	0	0	237671
2049	0	0	259330
2050	0	0	253025

EU·TOPIA

Table 8c. Screening colonoscopies per year and simulated scenario. Age >74 and period 2015-2050

Year	Scenario 1	Scenario 2	Scenario 3
2015	0	0	0
2016	0	0	0
2017	0	0	0
2018	0	0	0
2019	0	0	0
2020	0	0	0
2021	0	0	0
2022	0	0	0
2023	0	0	0
2024	0	0	0
2025	0	0	0
2026	0	0	0
2027	0	0	0
2028	0	0	0
2029	0	0	0
2030	0	0	0
2031	0	0	0
2032	0	0	0
2033	0	0	0
2034	0	0	0
2035	0	0	0
2036	0	0	0
2037	0	0	0
2038	0	0	136918
2039	0	0	130706
2040	0	0	139076
2041	0	0	141619
2042	0	0	145410
2043	0	0	146262
2044	0	0	130177
2045	0	0	140858
2046	0	0	137243
2047	0	0	135651
2048	0	0	138515
2049	0	0	112944
2050	0	0	111355

EU·TOPIA

Table 9a. Positive screening tests per year and simulated scenario. Age 40-49 and period 2015-2050

Year	Scenario 1	Scenario 2	Scenario 3
2015	0	0	0
2016	0	0	0
2017	0	0	0
2018	0	0	0
2019	0	0	0
2020	0	0	0
2021	0	0	0
2022	0	0	0
2023	0	0	0
2024	0	0	0
2025	0	0	0
2026	0	0	0
2027	0	0	0
2028	0	0	0
2029	0	0	0
2030	0	0	0
2031	0	0	0
2032	0	0	0
2033	0	0	0
2034	0	0	0
2035	0	0	0
2036	0	0	0
2037	0	0	0
2038	0	0	0
2039	0	0	0
2040	0	0	0
2041	0	0	0
2042	0	0	0
2043	0	0	0
2044	0	0	0
2045	0	0	0
2046	0	0	0
2047	0	0	0
2048	0	0	0
2049	0	0	0
2050	0	0	0

EU·TOPIA

Table 9b. Positive screening tests per year and simulated scenario. Age 50-74 and period 2015-2050

Year	Scenario 1	Scenario 2	Scenario 3
2015	0	37556	37556
2016	0	59075	59075
2017	0	39349	39349
2018	0	98003	446735
2019	0	41259	607233
2020	0	83433	59006
2021	0	47404	58822
2022	0	76418	61954
2023	0	47342	61437
2024	0	65270	55862
2025	0	50908	58922
2026	0	68057	58789
2027	0	50636	57218
2028	0	62110	172835
2029	0	54496	249756
2030	0	60331	78014
2031	0	55307	77276
2032	0	53484	79208
2033	0	58667	78697
2034	0	53825	75219
2035	0	52418	79565
2036	0	50745	77073
2037	0	51424	75564
2038	0	50998	74395
2039	0	49854	73469
2040	0	50136	71646
2041	0	51045	71888
2042	0	48251	71491
2043	0	48585	72828
2044	0	49615	77256
2045	0	50209	80784
2046	0	49844	78239
2047	0	49458	77714
2048	0	50432	74752
2049	0	51796	84422
2050	0	48741	82246

Table 9c. Positive screening tests per year and simulated scenario. Age >74 and period 2015-2050

Year	Scenario 1	Scenario 2	Scenario 3
2015	0	0	0
2016	0	0	0
2017	0	0	0
2018	0	0	0
2019	0	0	0
2020	0	6773	0
2021	0	0	0
2022	0	0	0
2023	0	0	0
2024	0	4729	0
2025	0	0	0
2026	0	0	0
2027	0	0	0
2028	0	5467	0
2029	0	0	0
2030	0	0	0
2031	0	0	0
2032	0	3945	0
2033	0	0	0
2034	0	0	0
2035	0	5112	0
2036	0	5145	0
2037	0	5074	0
2038	0	4465	36332
2039	0	4946	34793
2040	0	4924	36929
2041	0	4846	36645
2042	0	5015	38990
2043	0	5094	38422
2044	0	4785	34205
2045	0	5343	38081
2046	0	5145	37749
2047	0	4896	36412
2048	0	4847	37170
2049	0	3486	30348
2050	0	4173	29457

EU·TOPIA

Table 10a. Diagnostic follow-up colonoscopies performed per year and simulated scenario. Age 40-49 and period 2015-2050

Year	Scenario 1	Scenario 2	Scenario 3
2015	0	0	0
2016	0	0	0
2017	0	0	0
2018	0	0	0
2019	0	0	0
2020	0	0	0
2021	0	0	0
2022	0	0	0
2023	0	0	0
2024	0	0	0
2025	0	0	0
2026	0	0	0
2027	0	0	0
2028	0	0	0
2029	0	0	0
2030	0	0	0
2031	0	0	0
2032	0	0	0
2033	0	0	0
2034	0	0	0
2035	0	0	0
2036	0	0	0
2037	0	0	0
2038	0	0	0
2039	0	0	0
2040	0	0	0
2041	0	0	0
2042	0	0	0
2043	0	0	0
2044	0	0	0
2045	0	0	0
2046	0	0	0
2047	0	0	0
2048	0	0	0
2049	0	0	0
2050	0	0	0

Table 10b. Diagnostic follow-up colonoscopies performed per year and simulated scenario. Age 50-74 and period 2015-2050

Year	Scenario 1	Scenario 2	Scenario 3
2015	0	31823	31823
2016	0	50099	50099
2017	0	33433	33433
2018	0	83578	0
2019	0	34763	0
2020	0	71763	0
2021	0	40449	0
2022	0	64906	0
2023	0	40823	0
2024	0	55692	0
2025	0	42766	0
2026	0	58395	0
2027	0	43260	0
2028	0	53160	0
2029	0	45983	0
2030	0	50383	0
2031	0	46901	0
2032	0	45492	0
2033	0	49431	0
2034	0	45849	0
2035	0	44664	0
2036	0	43104	0
2037	0	43547	0
2038	0	42945	0
2039	0	42453	0
2040	0	43533	0
2041	0	43263	0
2042	0	41542	0
2043	0	41482	0
2044	0	42295	0
2045	0	42901	0
2046	0	41920	0
2047	0	42209	0
2048	0	42787	0
2049	0	43224	0
2050	0	41507	0

EU·TOPIA

Table 10c. Diagnostic follow-up colonoscopies performed per year and simulated scenario. Age >74 and period 2015-2050

Year	Scenario 1	Scenario 2	Scenario 3
2015	0	0	0
2016	0	0	0
2017	0	0	0
2018	0	0	0
2019	0	0	0
2020	0	5883	0
2021	0	0	0
2022	0	0	0
2023	0	0	0
2024	0	4015	0
2025	0	0	0
2026	0	0	0
2027	0	0	0
2028	0	4812	0
2029	0	0	0
2030	0	0	0
2031	0	0	0
2032	0	3353	0
2033	0	0	0
2034	0	0	0
2035	0	4409	0
2036	0	4483	0
2037	0	4218	0
2038	0	3848	0
2039	0	4175	0
2040	0	4161	0
2041	0	3903	0
2042	0	4230	0
2043	0	4317	0
2044	0	4013	0
2045	0	4438	0
2046	0	4216	0
2047	0	4113	0
2048	0	4061	0
2049	0	2941	0
2050	0	3499	0

Table 11a. False positive (%) per year and simulated scenario. Age 40-49 and period 2015-2050

Year	Scenario 1	Scenario 2	Scenario 3
2015	0	0	0
2016	0	0	0
2017	0	0	0
2018	0	0	0
2019	0	0	0
2020	0	0	0
2021	0	0	0
2022	0	0	0
2023	0	0	0
2024	0	0	0
2025	0	0	0
2026	0	0	0
2027	0	0	0
2028	0	0	0
2029	0	0	0
2030	0	0	0
2031	0	0	0
2032	0	0	0
2033	0	0	0
2034	0	0	0
2035	0	0	0
2036	0	0	0
2037	0	0	0
2038	0	0	0
2039	0	0	0
2040	0	0	0
2041	0	0	0
2042	0	0	0
2043	0	0	0
2044	0	0	0
2045	0	0	0
2046	0	0	0
2047	0	0	0
2048	0	0	0
2049	0	0	0
2050	0	0	0

EU·TOPIA

Table 11b. False positive (%) per year and simulated scenario. Age 50-74 and period 2015-2050

Year	Scenario 1	Scenario 2	Scenario 3
2015	0	3.3	3.3
2016	0	3.6	3.6
2017	0	2.9	2.9
2018	0	3.3	0
2019	0	2.4	0
2020	0	2.9	0
2021	0	2.4	0
2022	0	2.5	0
2023	0	2.4	0
2024	0	2.3	0
2025	0	2.2	0
2026	0	2.4	0
2027	0	2.1	0
2028	0	2.3	0
2029	0	2.1	0
2030	0	2.2	0
2031	0	2.2	0
2032	0	2.2	0
2033	0	2.1	0
2034	0	2.2	0
2035	0	2.1	0
2036	0	2.1	0
2037	0	2.2	0
2038	0	2.2	0
2039	0	2.1	0
2040	0	2.2	0
2041	0	2.2	0
2042	0	2.1	0
2043	0	2.1	0
2044	0	2.2	0
2045	0	2.2	0
2046	0	2.2	0
2047	0	2.2	0
2048	0	2.2	0
2049	0	2.2	0
2050	0	2.1	0

EU·TOPIA

Table 11c. False positive (%) per year and simulated scenario. Age >74 and period 2015-2050

Year	Scenario 1	Scenario 2	Scenario 3
2015	0	0	0
2016	0	0	0
2017	0	0	0
2018	0	0	0
2019	0	0	0
2020	0	4.6	0
2021	0	0	0
2022	0	0	0
2023	0	0	0
2024	0	2.8	0
2025	0	0	0
2026	0	0	0
2027	0	0	0
2028	0	2.4	0
2029	0	0	0
2030	0	0	0
2031	0	0	0
2032	0	1.8	0
2033	0	0	0
2034	0	0	0
2035	0	2	0
2036	0	2.1	0
2037	0	1.7	0
2038	0	1.5	0
2039	0	2.2	0
2040	0	1.7	0
2041	0	1.6	0
2042	0	1.9	0
2043	0	1.8	0
2044	0	1.9	0
2045	0	2	0
2046	0	1.9	0
2047	0	1.9	0
2048	0	1.9	0
2049	0	1.7	0
2050	0	2	0

Table 12a. Surveillance colonoscopies per year and simulated scenario. Age 40-49 and period 2015-2050

Year	Scenario 1	Scenario 2	Scenario 3
2015	0	0	0
2016	0	0	0
2017	0	0	0
2018	0	0	0
2019	0	0	0
2020	0	0	0
2021	0	0	0
2022	0	0	0
2023	0	0	0
2024	0	0	0
2025	0	0	0
2026	0	0	0
2027	0	0	0
2028	0	0	0
2029	0	0	0
2030	0	0	0
2031	0	0	0
2032	0	0	0
2033	0	0	0
2034	0	0	0
2035	0	0	0
2036	0	0	0
2037	0	0	0
2038	0	0	0
2039	0	0	0
2040	0	0	0
2041	0	0	0
2042	0	0	0
2043	0	0	0
2044	0	0	0
2045	0	0	0
2046	0	0	0
2047	0	0	0
2048	0	0	0
2049	0	0	0
2050	0	0	0

EU·TOPIA

Table 12b. Surveillance colonoscopies per year and simulated scenario. Age 50-74 and period 2015-2050

Year	Scenario 1	Scenario 2	Scenario 3
2015	0	0	0
2016	0	0	0
2017	0	5763	5763
2018	0	8018	7979
2019	0	9535	9416
2020	0	13695	19574
2021	0	24768	55712
2022	0	16816	74667
2023	0	22050	26215
2024	0	25491	146097
2025	0	21709	214310
2026	0	28311	43247
2027	0	29677	61235
2028	0	27367	78700
2029	0	30312	49371
2030	0	31333	106754
2031	0	28602	178593
2032	0	32003	73060
2033	0	32535	71679
2034	0	29867	97671
2035	0	30188	100113
2036	0	30572	97502
2037	0	31157	100539
2038	0	30665	1e+05
2039	0	29290	97530
2040	0	30160	96245
2041	0	28999	95512
2042	0	27560	91977
2043	0	28657	93596
2044	0	27144	91029
2045	0	25542	90832
2046	0	26240	88648
2047	0	26731	86040
2048	0	26105	85699
2049	0	26114	86032
2050	0	25682	87346

EU·TOPIA

Table 12c. Surveillance colonoscopies per year and simulated scenario. Age >74 and period 2015-2050

Year	Scenario 1	Scenario 2	Scenario 3
2015	0	0	0
2016	0	0	0
2017	0	0	0
2018	0	0	0
2019	0	1139	1139
2020	0	1200	2612
2021	0	1189	7299
2022	0	2228	8343
2023	0	2549	3445
2024	0	3174	168
2025	0	2629	1802
2026	0	2235	1105
2027	0	2299	6478
2028	0	2805	6933
2029	0	1819	316
2030	0	2747	953
2031	0	2287	1839
2032	0	3630	3054
2033	0	2167	6124
2034	0	2082	5735
2035	0	4942	12426
2036	0	4590	43
2037	0	4068	20913
2038	0	4380	149
2039	0	3747	128
2040	0	4379	65
2041	0	4407	66
2042	0	5124	109
2043	0	4015	43
2044	0	4270	64
2045	0	4934	108
2046	0	4367	43
2047	0	4439	87
2048	0	4760	44
2049	0	3965	87
2050	0	3390	152

Table 13a. Colonoscopy complications per year and simulated scenario. Age 40-49 and period 2015-2050

Year	Scenario 1	Scenario 2	Scenario 3
2015	0	0	0
2016	0	0	0
2017	0	0	0
2018	0	0	0
2019	0	0	0
2020	0	0	0
2021	0	0	0
2022	0	0	0
2023	0	0	0
2024	0	0	0
2025	0	0	0
2026	0	0	0
2027	0	0	0
2028	0	0	0
2029	0	0	0
2030	0	0	0
2031	0	0	0
2032	0	0	0
2033	0	0	0
2034	0	0	0
2035	0	0	0
2036	0	0	0
2037	0	0	0
2038	0	0	0
2039	0	0	0
2040	0	0	0
2041	0	0	0
2042	0	0	0
2043	0	0	0
2044	0	0	0
2045	0	0	0
2046	0	0	0
2047	0	0	0
2048	0	0	0
2049	0	0	0
2050	0	0	0

EU·TOPIA

Table 13b. Colonoscopy complications per year and simulated scenario. Age 50-74 and period 2015-2050

Year	Scenario 1	Scenario 2	Scenario 3
2015	0	195	195
2016	0	420	420
2017	0	274	274
2018	0	747	5030
2019	0	286	6075
2020	0	646	504
2021	0	436	751
2022	0	586	874
2023	0	391	529
2024	0	563	1147
2025	0	416	1429
2026	0	593	570
2027	0	458	704
2028	0	554	2524
2029	0	502	3254
2030	0	553	1231
2031	0	493	1663
2032	0	505	1041
2033	0	549	1032
2034	0	491	1193
2035	0	491	1216
2036	0	496	1208
2037	0	499	1186
2038	0	502	1200
2039	0	482	1124
2040	0	493	1117
2041	0	485	1116
2042	0	453	1094
2043	0	461	1114
2044	0	462	1114
2045	0	441	1138
2046	0	451	1103
2047	0	443	1086
2048	0	440	1049
2049	0	436	1136
2050	0	429	1133

EU·TOPIA

Table 13c. Colonoscopy complications per year and simulated scenario. Age >74 and period 2015-2050

Year	Scenario 1	Scenario 2	Scenario 3
2015	0	0	0
2016	0	0	0
2017	0	0	0
2018	0	0	0
2019	0	13	13
2020	0	116	31
2021	0	16	90
2022	0	23	97
2023	0	28	29
2024	0	95	1
2025	0	34	23
2026	0	30	16
2027	0	28	84
2028	0	93	92
2029	0	24	4
2030	0	33	13
2031	0	31	26
2032	0	82	34
2033	0	28	73
2034	0	27	75
2035	0	105	98
2036	0	105	0
2037	0	94	191
2038	0	83	791
2039	0	96	756
2040	0	89	802
2041	0	89	795
2042	0	106	847
2043	0	94	834
2044	0	94	743
2045	0	106	827
2046	0	99	819
2047	0	95	791
2048	0	101	807
2049	0	80	659
2050	0	83	640

EU·TOPIA

Table 14a. Adenomas detected by screening per year and simulated scenario. Age 40-49 and period 2015-2050

Year	Scenario 1	Scenario 2	Scenario 3
2015	0	0	0
2016	0	0	0
2017	0	0	0
2018	0	0	0
2019	0	0	0
2020	0	0	0
2021	0	0	0
2022	0	0	0
2023	0	0	0
2024	0	0	0
2025	0	0	0
2026	0	0	0
2027	0	0	0
2028	0	0	0
2029	0	0	0
2030	0	0	0
2031	0	0	0
2032	0	0	0
2033	0	0	0
2034	0	0	0
2035	0	0	0
2036	0	0	0
2037	0	0	0
2038	0	0	0
2039	0	0	0
2040	0	0	0
2041	0	0	0
2042	0	0	0
2043	0	0	0
2044	0	0	0
2045	0	0	0
2046	0	0	0
2047	0	0	0
2048	0	0	0
2049	0	0	0
2050	0	0	0

EU·TOPIA

Table 14b. Adenomas detected by screening per year and simulated scenario. Age 50-74 and period 2015-2050

Year	Scenario 1	Scenario 2	Scenario 3
2015	0	19614	19614
2016	0	31729	31729
2017	0	20545	20545
2018	0	50883	322756
2019	0	19642	434783
2020	0	42151	41000
2021	0	22551	40897
2022	0	36673	43139
2023	0	23206	42628
2024	0	30690	39310
2025	0	23154	41308
2026	0	31702	41150
2027	0	22783	39710
2028	0	28951	102822
2029	0	24603	140666
2030	0	27276	48861
2031	0	24805	48234
2032	0	24208	49709
2033	0	25663	48750
2034	0	23885	47288
2035	0	22997	50545
2036	0	23141	49025
2037	0	23584	47438
2038	0	23632	46410
2039	0	22745	46433
2040	0	23100	45094
2041	0	22760	46258
2042	0	21524	45337
2043	0	21776	46569
2044	0	21835	49160
2045	0	23101	52552
2046	0	22300	49902
2047	0	22307	50170
2048	0	22345	47174
2049	0	22933	54852
2050	0	21686	53460

Table 14c. Adenomas detected by screening per year and simulated scenario. Age >74 and period 2015-2050

Year	Scenario 1	Scenario 2	Scenario 3
2015	0	0	0
2016	0	0	0
2017	0	0	0
2018	0	0	0
2019	0	0	0
2020	0	4199	0
2021	0	0	0
2022	0	0	0
2023	0	0	0
2024	0	2585	0
2025	0	0	0
2026	0	0	0
2027	0	0	0
2028	0	2375	0
2029	0	0	0
2030	0	0	0
2031	0	0	0
2032	0	1499	0
2033	0	0	0
2034	0	0	0
2035	0	2109	0
2036	0	2177	0
2037	0	1713	0
2038	0	1382	19511
2039	0	2355	18752
2040	0	1786	19821
2041	0	1644	18723
2042	0	2028	20947
2043	0	2008	20155
2044	0	1802	18024
2045	0	2111	20833
2046	0	1989	20927
2047	0	1937	19497
2048	0	2184	20062
2049	0	1525	16380
2050	0	1891	15626

Table 15a. Colorectal cancers detected by screening per year and simulated scenario. Age 40-49 and period 2015-2050

Year	Scenario 1	Scenario 2	Scenario 3
2015	0	0	0
2016	0	0	0
2017	0	0	0
2018	0	0	0
2019	0	0	0
2020	0	0	0
2021	0	0	0
2022	0	0	0
2023	0	0	0
2024	0	0	0
2025	0	0	0
2026	0	0	0
2027	0	0	0
2028	0	0	0
2029	0	0	0
2030	0	0	0
2031	0	0	0
2032	0	0	0
2033	0	0	0
2034	0	0	0
2035	0	0	0
2036	0	0	0
2037	0	0	0
2038	0	0	0
2039	0	0	0
2040	0	0	0
2041	0	0	0
2042	0	0	0
2043	0	0	0
2044	0	0	0
2045	0	0	0
2046	0	0	0
2047	0	0	0
2048	0	0	0
2049	0	0	0
2050	0	0	0

Table 15b. Colorectal cancers detected by screening per year and simulated scenario. Age 50-74 and period 2015-2050

Year	Scenario 1	Scenario 2	Scenario 3
2015	0	2083	2083
2016	0	4105	4105
2017	0	1970	1970
2018	0	6690	8498
2019	0	1617	9516
2020	0	5259	632
2021	0	2258	496
2022	0	4166	768
2023	0	1679	720
2024	0	3237	637
2025	0	2336	502
2026	0	3511	638
2027	0	2312	776
2028	0	3016	2349
2029	0	2838	3606
2030	0	2825	923
2031	0	2829	1004
2032	0	2742	960
2033	0	2860	983
2034	0	2623	969
2035	0	2461	830
2036	0	2381	777
2037	0	2436	950
2038	0	2631	894
2039	0	2454	854
2040	0	2833	897
2041	0	2192	895
2042	0	2336	715
2043	0	2519	860
2044	0	2759	870
2045	0	2308	1010
2046	0	2545	1129
2047	0	2358	996
2048	0	2437	1057
2049	0	1959	1097
2050	0	2418	985

EU·TOPIA

Table 15c. Colorectal cancers detected by screening per year and simulated scenario. Age >74 and period 2015-2050

Year	Scenario 1	Scenario 2	Scenario 3
2015	0	0	0
2016	0	0	0
2017	0	0	0
2018	0	0	0
2019	0	0	0
2020	0	503	0
2021	0	0	0
2022	0	0	0
2023	0	0	0
2024	0	231	0
2025	0	0	0
2026	0	0	0
2027	0	0	0
2028	0	430	0
2029	0	0	0
2030	0	0	0
2031	0	0	0
2032	0	217	0
2033	0	0	0
2034	0	0	0
2035	0	277	0
2036	0	299	0
2037	0	321	0
2038	0	340	446
2039	0	257	428
2040	0	283	479
2041	0	307	832
2042	0	371	719
2043	0	345	711
2044	0	408	557
2045	0	431	517
2046	0	368	626
2047	0	348	761
2048	0	175	611
2049	0	174	522
2050	0	130	499
