# Supplementary material for: The EU-TOPIA evaluation tool: An online modelling-based tool for informing breast, cervical, and colorectal cancer screening decisions in Europe
Source: Prev Med Rep. 2021 Apr 30;22:101392. doi: 10.1016/j.pmedr.2021.101392 (PMC8122113; doi:10.1016/j.pmedr.2021.101392)
Supplement: Supplementary data 5 [file mmc5.docx]

**Model adjustment based on the user’s data**

As described above, users can verify and select which data to use for simulating with the EU-TOPIA evaluation tool. This section provides a brief overview of the adjustments that automatically convert the EU-TOPIA regional models with the data provided and selected by the user. Those adjustments are divided in three groups: i) adjustments that directly incorporate user’s data into the model (“direct”); ii) adjustments that indirectly incorporate user’s data into the model (“indirect”); and iii) elaborations that adjust model parameters comparing the European regional models (Chapter 4 and General Appendix) and the specific data provided by the users (“elaboration”). **Supplementary Table 2** provides an overview of the model adjusted parameters, data source, and type of adjustment.

**Supplementary Table 2**. Overview of the adjustments for the model parameters.

| Model parameters (Figure 1.6) | Data from the  excel template (Table 1.1) | Type  of adjustment |
| --- | --- | --- |
| Cancer incidence | eTable2 | Elaboration |
| Screening coverage/invitation | sTable1a  sTable1b  sTable2 | Direct/Indirect |
| Population size | eTable1  eTable6 | Indirect |
| Cancer mortality | eTable3 | - |
| Cancer specific survival | eTable4 | Elaboration |
| Stage distribution | eTable5 | - |
| All-cause mortality | eTable6 | Indirect |
| Cancer specific localization | eTable7 | Direct |
| Screening history | sTable3a | Indirect |
| Screening outcomes | sTable1a  sTable4  sTable5  sTable7a  sTable7b | Indirect\  Elaboration |
| Participation in follow-up  colonoscopy | sTable5 | Direct |
| Completation follow-up  colonoscopy | sTable6a  sTable6b | Direct |
| Complication at diagnostic  colonoscopy | sTable9 | Direct |
| Interval cancers | sTable11 | - |
| Post-colonoscopy recommendations | sTable1a | Direct |

Cancer incidence

Cancer incidence data are collected from eTable2 (excel template). This information is mandatory (users are not allowed to simulate if they do not provide this data). The number of cases and population size per age group and gender are aggregated and as shown in **Supplementary Table 3**. According to the region of the user’s country (data provided in eTable0, excel template), the data are compared with those reported by the regional exemplary country in the pre-screening period (General Appendix; Eastern Europe, Slovenia 2004-2008; Northern Europe, Finland 1999-2003; Southern Europe, Italy 1998-2002; and Western Europe, The Netherlands, 1999-2003).

The comparison is done estimating a standardized incidence ratio, taking in consideration the population aged 35 years-old or older (SIR; **Supplementary Table 3**). The SIR is incorporated in the model for adjusting incidence parameters (across all ages) of the regional exemplary country model (**Supplementary Figure 11**). In the case of the CRC version of the EU-TOPIA evaluation tool, those incidence parameters are the parameters for the onset of adenomas (**General Appendix, Appendix Figure 2**, arrow from state 1 [No lesion] and state 2 [Adenoma <5mm]), which are the driving parameters that affect the CRC incidence in the model.

**Supplementary Table 3**. Example of cancer incidence adjustment and model standard incidence ratio

| User’s country data | | | | | Regional model (A) | | | |
| --- | --- | --- | --- | --- | --- | --- | --- | --- |
| Age | No. CRCs  (Observed) | Pop. size | Rate  (x100,000  Person-years) | Rate (A) *  Pop. Size (Expected) | No.  CRCs (A) | Pop.  Size (A) | Rate  (A) |  |
| 0-19 | 66 | 6877817 | 0.62590027 | - | - | - | - |  |
| 20-24 | 50 | 1836535 | 2.26298956 | - | - | - | - |  |
| 25-29 | 51 | 1927458 | 2.67691858 | - | - | - | - |  |
| 30-34 | 94 | 1952204 | 4.16514707 | - | - | - | - |  |
| 35-39 | 574 | 9053758 | 6.3399088 | 485.6043338 | 351 | 6544153 | 5.36357E-05 |  |
| 40-44 | 1150 | 7966466 | 14.43551 | 887.4148515 | 677 | 6077538 | 0.000111394 |  |
| 45-49 | 2240 | 7414738 | 30.2101032 | 1736.20528 | 1337 | 5709869 | 0.000234156 |  |
| 50-54 | 4134 | 7535836 | 54.8578817 | 3819.244514 | 2865 | 5652995 | 0.000506811 |  |
| 55-59 | 6168 | 6855658 | 89.9694821 | 5922.000249 | 3744 | 4334276 | 0.000863812 |  |
| 60-64 | 9214 | 6738458 | 136.737515 | 9632.670368 | 5184 | 3626426 | 0.001429507 |  |
| 65-69 | 11850 | 6067956 | 195.288166 | 12226.87056 | 6484 | 3217882 | 0.00201499 |  |
| 70-74 | 14266 | 5535568 | 257.715197 | 15196.80343 | 7607 | 2770916 | 0.002745302 |  |
| 75-79 | 14324 | 4578144 | 312.877882 | 15842.49582 | 7750 | 2239585 | 0.003460463 |  |
| 80-84 | 8712 | 2413192 | 361.015617 | 9299.676031 | 5453 | 1415010.15 | 0.003853683 |  |
| ≥ 85 | 8902 | 2578446 | 345.246711 | 8606.659914 | 3725 | 1115962.69 | 0.003337925 |  |
| Total | 81534 | - | - | 83655.64536 | - | - | - |  |
| SIR (O/E) | 0.974638 |  |  |  |  |  |  |  |

**Supplementary Figure 11**. Example of cancer incidence adjustment

Screening coverage/invitation

In this data category, information is directly or indirectly incorporated in the model. The EU-TOPIA evaluation tool directly incorporates the information included in the current screening table (sTable1a, excel template), such as year of starting screening, roll-out period (completing year), screening test(s), target age(s), and screening interval(s). Moreover, the proportion of not invited individuals and the rate of screening participation among those invited are also directly incorporated in this category (from sTable2, excel template). The first proportion is computed aggregating data for both males and females (from cells 30 and 61, column E; % of invited, *invitation coverage*) and computing its complementary to one (1-% of invited = % not invited). The rate of screening participation among invited individuals is computed considering information on participation rate (aggregated for both males and females) from lines 15 and 46 (column J). That rate is assumed across all target ages (age-specific participation rates cannot be modelled in the CRC version of the EU-TOPIA evaluation tool).

Given the % of invited individuals in screening, the model stratified these individuals in two different groups: those that will attend at least one time screening (ever screened); and those who will never attend (never screened). The division is performed using the stratification provided in **Supplementary Table 4**. For example, if user provides data with a participation rate of 45%, the model will assume that 75% of the invited individuals could attend at least one screening round and 25% of the invited will never attend screening. Those that attend screening are assumed to have an overall CRC risk 1.7% lower that the not invited group, whereas those who never attend were assumed to have a 15% higher risk of developing CRC compared to the not invited group. The proportion of individuals that never attended screening was assumed to be at least 10%.

**Supplementary Table 4**. Assumptions for invited individuals

| Participation rate (sTable 2) | % of invited *“ever screened”* | % of invited *“never screened”* |
| --- | --- | --- |
| ≥70% | 90% | 10% |
| [60%; 70%) | 85% | 15% |
| [50%; 60%) | 80% | 20% |
| [40%; 50%) | 75% | 25% |
| <40% | 70% | 30% |

After computing the proportion of invited individuals that could attend at least one screening round, in the model participation rate will be adjusted to take in account this stratification as follow:

$${Participation}_{Adjusted}= \frac{{Participation}_{Not-Adjusted}}{\frac{\% Invited "ever screened"}{\left( \% Invited "\text{ever screened}\text{"}+\% Invited "never screened" \right)}}$$

And it will be assumed only in the group of Invited individuals “*ever screened*”. Among individuals invited that never attend screening, participation rate was assumed as for the not invited group and equals to zero.

Population size and all-cause mortality

The model replicates the age-specific population provided by the user in eTable1 (excel template) for year 2015. As described in General Appendix, MISCAN simulates the life-history of each individual for birth to death. This simulation is performed simulating and following several specific cohorts from birth to death. MISCAN needs, therefore, to incorporate information of those who born and died before 2015 for replicating correctly the age-specific country population. The model, combining information on population size (eTable1, excel template) and all-cause mortality (eTable6, excel template), is able to include that missing information following a Lexis diagram. Moreover, data on all-cause mortality (eTable6, age-specific rates, $r_{a}$) is indirectly incorporate in the model as cumulative probability (P). This indirect incorporation is performed with the following formulas for:

Age-specific mortality probability (p) for age a:

$p_{a}=1-e^{-r_{a}}$

Age-specific mortality cumulative probability (P) for age a:

$P_{a}=\left\{ \begin{aligned} \begin{matrix} p_{a} & a=0 \end{matrix} \\ \\ \begin{matrix} P_{a-1}+\left( 1-P_{a-1} \right)p_{a} & a>0 \end{matrix} \end{aligned} \right.$

The information provided in eTable6 (all-cause mortality) is assumed for each simulated year. No dynamic changes in all-cause mortality rates could be assumed in the CRC version of the EU-TOPIA evaluation tool.

Cancer mortality, stage distribution, and interval cancers

In the CRC version of the EU-TOPIA evaluation tool, data on CRC mortality, stage distribution, and interval CRCs are not used by the model. CRC mortality (eTable3) is not necessary because it would led to overfitting the model. Considering both data on CRC incidence and CRC relative survival, the model is already able to replicate the entire natural history of CRC (please see results provided in Chapter 4). CRC stage distribution is not incorporated because it is not possible, unfortunately, to incorporate any feasible model adjustment that permits the model to replicate the country-specific CRC stage distribution. Those parameters need to be calibrated, but an user-specific calibration inside the tool is not feasible. Thus, in each model simulation, the model uses the CRC stage distribution parameters of the corresponding regional exemplary country model. Finally, data on interval CRCs are not included in the model because the EU-TOPIA evaluation tool is not currently programmed to estimate interval cancer results.

Cancer-specific survival

As described in Gini et al, once the model simulates a clinical diagnosis of CRC, an individual age-, stage-, and localization-specific CRC relative survival is set inside the model. Our model defined tow components in that phase: i) an age-, stage-, and localization-specific survival time (part A), and ii) the corresponding event at the end of that defined time (CRC death or no-CRC death). The Event is defined considering specific age-, stage-, and localization-specific probabilities (part B). In the Dutch MISCAN-Colon model version, those probabilities were informed with the age-, stage-, and localization-specific observed cumulative mortality rates for 2010-2014 (5-years CRC relative survival, respectively, 64 for colon and 67% for rectum cancer). Survival times were included in the model using a piece-wise distribution, based on the time-specific distribution of CRC deaths (up to a max of 15 years of follow-up since the CRC diagnosis) in the Dutch Cancer Registry.

In the CRC version of the EU-TOPIA evaluation tool, the model adjust CRC relative survival based on the user’s information (eTable4, cell I12, excel template). The adjustment is performed as follow: i) the model assumed the same Dutch piece-wise age-, stage-, and localization-specific distributions for simulating the survival time (part A); ii) the model compared the 5-year CRC relative survival provided by the user (eTable4, cell I12, excel template) and the Dutch 5-year CRC relative survival observed in 2010-2014 (computing a ratio); and iii) the model adjusted the Dutch each age-, stage-, and localization-specific event probabilities (observed cumulative mortality rates for the period 2010-2014; part B), applying for each of those probabilities the previously computed ratio.

Cancer specific localization, participation in follow-up colonoscopy, completation follow-up colonoscopy, complication at diagnostic colonoscopy, and post-colonoscopy recommendations

Data included in this section were incorporated directly in the model. Briefly, those information (as collected in the excel template) were directly inserted in the model without requiring any adjustment.

Screening history

Screening history in MISCAN-Colon is characterized by two important model parameters: i) probability of attending screening if previous screening round was attended (APY); and ii) probability of attending screening if previous screening round was not attended (APN). In the CRC version of the EU-TOPIA evaluation tool, this information is collected in sTable3a (Boxes: C6.M, C7.M, C8.M, C6.W, C7.W, C8.W, excel template). The total values computed in lines 32 and 82 (of sTable3a, excel template) were aggregated per gender, summing up data from males and females. Using the aggregated data, we computed the proportion of screened individual that attended the previous screening round (PrY) and the proportion of screened individual that did not attend the previous screening round (PrN). Then, we computed the proportion of the participation rate (sTable2, excel template; participation rate not adjusted) due to those that, respectively, attended (%Participation-PrY) and did not attend (%Participation-PrN) the previous screening round as:

$${\%Participation}_{PrY}={Participation}_{Not-Adjusted}*PrY$$

$${\%Participation}_{PrN}={Participation}_{Not-Adjusted}*PrN$$

Finally, we computed the probability of attending screening given that the previous screening round was attended (APY) as:

$$APY= \frac{{\%Participation}_{PrY}}{{Participation}_{Not-Adjusted}} ;$$

And the probability of attending screening given that the previous screening round was not attended (APN) as:

$$APN= \frac{{\%Participation}_{PrN}}{\left( 1-{Participation}_{Not-Adjusted} \right)} .$$

These values were elaborated assuming that screening participation rates are constant across all the screening rounds (except when selected by the user inside the EU-TOPIA evaluation tool). Thus, our tool assumes this equilibrium:

$${Participation}_{Not-Adjusted}\left( {round}_{t} \right)=$$

$$APY*{Participation}_{Not-Adjusted}\left( {round}_{t-1} \right)+$$

$$APN*\left( 1-{Participation}_{Not-Adjusted}\left( {round}_{t-1} \right) \right);$$

$$for \forall t>1.$$

However, data collected by the users are year-specific and, therefore, it might be possible that those information violated this equilibrium (or using screening history data from an exemplary country violated this equilibrium). In those cases, the CRC version of EU-TOPIA evaluation tool recognizes and corrects the disequilibrium increasing or decreasing APY and APN accordingly.

Screening outcomes

This section provides information about the adjustment for screening specificity and sensitivity performed inside the CRC version of the EU-TOPIA evaluation. This adjustment is function of several information such as the test performed in the country (sTable1a, excel template), the number of positive tests (sTable4, excel template), the number of diagnostic examination performed (sTable5, excel template), and the number of lesion detected (sTable7a or sTable7b, excel template). Briefly, the tool adjusts the regional exemplary country model calibrated parameters (test sensitivity), taking as input differences in detection rates and SIR (CRC incidence section) between user’s country and corresponding regional exemplary country, as follow:

$${Sens}_{(User^{'}s data)}= {Sens}_{(Regional exemplary country)}* \left( \frac{\frac{{Dectection Rate for Adenomas}_{User^{'}s data}}{{Dectection Rate for Adenomas}_{Regional exemplary country}}}{SIR \left( see above \right)} \right).$$

Specificity was computed directly from the data (sTable4, Negatives; and sTabla7a or sTable7b, False Positive – No lesions detected, excel template). Adjustment for sensitivity was performed only when screening outcomes were collected for gFOBT or FIT screening. In case of gFOBT screening, Finnish model parameters were adjusted (regardless of the European region) because Finland is the only exemplary country with parameters calibrated for gFOBT. For FIT screening, sensitivities calibrated replicating the study of Imperiale et al are adjusted for Northern and Southern European countries. For Western and Eastern European countries, the tool will adjust values, respectively, calibrated for the Dutch and Slovenian MISCAN-Colon model version (N.B. Slovenian FIT sensitivities were calibrated considering 2-FIT sample – at least one positive). For FS and Colonoscopy screening, sensitivities were not adjusted and assumed fixed across European countries (75% for adenomas <5mm; 85% for adenoma 6-9mm; and 95% for large adenomas, >10mm, and CRCs).
